# Antiproliferative Phenanthrenes from *Juncus tenuis*: Isolation and Diversity-Oriented Semisynthetic Modification

**DOI:** 10.3390/molecules25245983

**Published:** 2020-12-17

**Authors:** Csaba Bús, Norbert Kúsz, Annamária Kincses, Nikoletta Szemerédi, Gabriella Spengler, László Bakacsy, Dragica Purger, Róbert Berkecz, Judit Hohmann, Attila Hunyadi, Andrea Vasas

**Affiliations:** 1 Department of Pharmacognosy, University of Szeged, 6720 Szeged, Hungary; bcsaba0312@gmail.com (C.B.); kusznorbert@gmail.com (N.K.); hohmann.judit@szte.hu (J.H.); 2 Department of Medical Microbiology and Immunobiology, University of Szeged, Dóm tér 10, 6720 Szeged, Hungary; kincses.annamaria90@gmail.com (A.K.); szemeredi.nikoletta@med.u-szeged.hu (N.S.); spengler.gabriella@med.u-szeged.hu (G.S.); 3 Department of Plant Biology, University of Szeged, Közép Fasor 52, 6726 Szeged, Hungary; bakacsy@gmail.com; 4 Department of Pharmacognosy, University of Pécs, Rókus u. 2, 7624 Pécs, Hungary; dragica@gamma.ttk.pte.hu; 5 Institute of Pharmaceutical Analysis, University of Szeged, Somogyi u. 4, 6720 Szeged, Hungary; berkecz.robert@pharm.u-szeged.hu; 6 Interdisciplinary Centre of Natural Products, University of Szeged, Eötvös u. 6, 6720 Szeged, Hungary

**Keywords:** *Juncus tenuis*, phenanthrene, juncusol, effusol, semisynthetic derivatives, antiproliferative activity

## Abstract

The occurrence of phenanthrenes is limited in nature, with such compounds identified only in some plant families. Phenanthrenes were described to have a wide range of pharmacological activities, and numerous research programs have targeted semisynthetic derivatives of the phenanthrene skeleton. The aims of this study were the phytochemical investigation of *Juncus tenuis*, focusing on the isolation of phenanthrenes, and the preparation of semisynthetic derivatives of the isolated compounds. From the methanolic extract of *J. tenuis*, three phenanthrenes (juncusol, effusol, and 2,7-dihydroxy-1,8-dimethyl-5-vinyl-9,10-dihydrophenanthrene) were isolated. Juncusol and effusol were transformed by hypervalent iodine(III) reagent, using a diversity-oriented approach. Four racemic semisynthetic compounds possessing an alkyl-substituted *p*-quinol ring (**1**–**4**) were produced. Isolation and purification of the compounds were carried out by different chromatographic techniques, and their structures were elucidated by means of 1D and 2D NMR, and HRMS spectroscopic methods. The isolated secondary metabolites and their semisynthetic analogues were tested on seven human tumor cell lines (A2780, A2780cis, KCR, MCF-7, HeLa, HTB-26, and T47D) and on one normal cell line (MRC-5), using the MTT assay. The effusol derivative **3**, substituted with two methoxy groups, showed promising antiproliferative activity on MCF-7, T47D, and A2780 cell lines with IC_50_ values of 5.8, 7.0, and 8.6 µM, respectively.

## 1. Introduction

To date, approximately 500 phenanthrenes from 59 genera of 18 families were isolated. The major sources of phenanthrenes are Orchidaceae, Juncaceae, Combretaceae, and Dioscoreaceae species [1,2]. The family Juncaceae includes up to 500 species belonging to seven genera (*Distichia*, *Juncus*, *Luzula*, *Marsippospermum*, *Oxychloe*, *Patosia*, and *Rostkovia*). Among them, *Juncus* is the largest one, which comprises approximately 340 species [3]. Besides flavonoids, phenanthrenes are the main bioactive constituents of the Juncaceae species [4]. To date, more than one hundred phenanthrenes have been isolated from ten *Juncus* species (*J. acutus*, *J. atratus*, *J. compressus*, *J. effusus*, *J. gerardii*, *J. inflexus*, *J. maritimus*, *J. roemerianus*, *J. setchuensis*, and *J. subulatus*) [5,6,7,8]. Some *Juncus* species are extremely rich sources of phenanthrenes; for example, from *J. effusus*, 58 compounds were reported in previous studies. Since various *Juncus* phenanthrenes are substituted with a vinyl group, such compounds are considered to be important chemotaxonomic markers of the genus [5,9].

Phenanthrenes have become the subjects of numerous pharmacological studies. Their antiproliferative, antimicrobial (antifungal, antiviral, and antibacterial), antialgal, anti-inflammatory, antioxidant, anxiolytic, and hepatoprotective activities were reported [2,10]. The presence of a quinone moiety in the molecules resulted in increased antiproliferative activity [11]. The cytotoxicity of quinones can be explained by many mechanisms, including intercalation, inhibition of DNA and RNA, by breaking of DNA strands, alteration of cell membrane functions, redox cycling that leads to the formation of reactive oxygen species (ROS), and ROS- or Michael-addition-mediated alkylation of various biochemical targets [12,13,14]. While quinones, in general, are considered as unfavorable to be included in screening libraries due to their tendency to behave as pan-assay interference compounds (PAINS) [15], many approved anticancer drugs have such a structure. Furthermore, the quinone and quinol moieties are not only versatile synthons to increase chemical diversity [16], but these may also serve as warheads for potential covalent inhibitors of well-defined biochemical targets [17,18]. Concerning further structure–activity relationships of phenanthrene derivatives, the combined presence of methyl, hydroxy, and vinyl substituents on ring C, a methyl and a hydroxy group on ring A, and a single bond between C-9 and C-10 increase the antiproliferative effect of phenanthrenes [19]. Denbinobin, isolated from an orchid species (*Dendrobium nobile*), is the most widely investigated phenanthrene quinone. It exerts cytotoxic activity through different mechanisms, like apoptosis induction via caspase-dependent and independent pathways [20,21,22], inducing oxidative stress through increasing ROS levels [23], and inhibition of the topoisomerase II enzyme [24]. Moreover, 5-hydroxy-2,3-dimethoxy-1,4-phenanthrenequinone showed specific cytotoxicity against the HL-60 cell line (with an IC_50_ value of 4.7 μM) [25]. Another phenanthraquinone, calanquinone A, was tested against several cancer cell lines. This compound inhibited the growth of A549 (IC_50_ 0.61 μM), PC-3 (IC_50_ 0.51 μM), DU145 (IC_50_ 1.08 μM), HCT-8 (IC_50_ 0.64 μM), MCF-7 (IC_50_ 0.10 μM), KB (IC_50_ 1.02 μM), and vincristine resistant KBVIN (IC_50_ 1.43 μM) cells, respectively [26]. The aims of our work were the isolation and structure determination of phenanthrenes from *Juncus tenuis*, the preparation of oxidized semisynthetic derivatives of the isolated phenanthrenes motivated by the aforementioned structural attributes of quinoidal compounds, and the characterization of the antiproliferative activity of these compounds on human adherent tumor cell lines. The value of the chosen semisynthetic approach in increasing the chemical–pharmacological diversity was previously demonstrated by our group. Starting from juncuenin B, we prepared and characterized eleven derivatives via hypervalent iodine-catalyzed oxidation. Among these differently substituted compounds bearing *p*- or *o*-quinol rings, three showed considerable antiproliferative effects against different tumor cell lines, and their activity was enantiospecific [27].

From the dichloromethane fraction of the methanolic extract of *J. tenuis*, three compounds (juncusol, effusol, and 2,7-dihydroxy-1,8-dimethyl-5-vinyl-9,10-dihydrophenanthrene) were isolated. As a result of the semisynthetic processes, two juncusol derivatives (**1**,**2**) and two effusol derivatives (**3**,**4**) were obtained. The structures of these compounds were elucidated by 1D and 2D NMR (Nuclear Magnetic Resonance), and MS (Mass Spectrometry) measurements. The antiproliferative activity of the compounds was investigated on seven human tumor cell lines (A2780, A2780cis, KCR, MCF-7, HeLa, HTB-26, and T47D) and on MRC-5 human embryonal lung fibroblasts.

## 2. Results and Discussion

### 2.1. Isolation, Semisynthetic Derivatization, and Structure Determination of the Compounds

Dried aerial parts of *J. tenuis* were ground and extracted with MeOH, at room temperature. After concentration, the extract was dissolved in 50% aqueous MeOH, and solvent–solvent partition was performed with *n*-hexane and CH_2_Cl_2_ (dichloromethane). The CH_2_Cl_2_ phase was separated and purified with a combination of different chromatographic methods (CC, VLC, RPC, MPLC, and HPLC) to afford three compounds.

The structure determination was carried out by extensive spectroscopic analysis, using 1D NMR (^1^H and JMOD) spectroscopy and a comparison of the spectral data with those published in the literature [28,29]. Based on the NMR spectra and the literature data, juncusol, effusol, and 2,7-dihydroxy-1,8-dimethyl-5-vinyl-9,10-dihydrophenanthrene were identified. *J. tenuis* is an abundant source of juncusol and effusol, as approximately 200 mg (0.012% *w*/*w*) were isolated from 1.68 kg of dried plant material.

Juncusol was isolated previously from numerous *Juncus* species (*J. acutus*, *J. effusus*, *J. inflexus*, *J. roemerianus*, *J. setchuensis*, and *J. subulatus*) [9,30,31,32,33,34]. Similarly to juncusol, effusol is also a ubiquitous phenanthrene in *Juncus* species, as it was isolated from *J. acutus*, *J. atratus*, *J. compressus*, *J. effusus*, *J. maritimus*, *J. setchuensis*, and *J. subulatus* [6,7,30,33,34,35,36]. Moreover, 2,7-Dihydroxy-1,8-dimethyl-5-vinyl-9,10-dihydrophenanthrene was identified before from *J. acutus* and *J. effusus* [19,28,29].

**Scheme 1:**
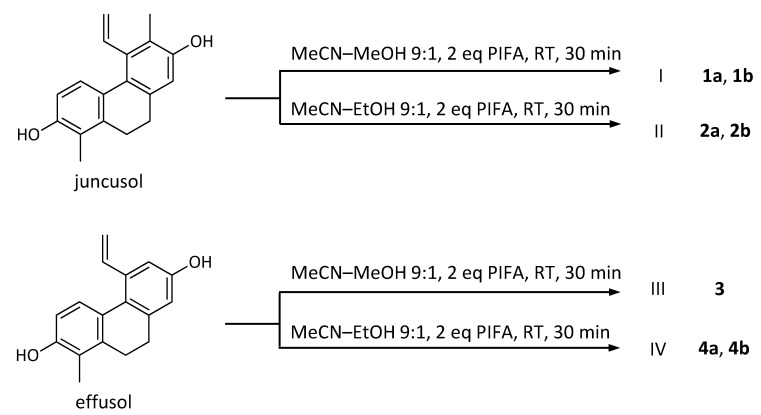
Transformation of juncusol and effusol under different conditions. PIFA: [bis-(trifluoroacetoxy)]iodobenzene.

**Figure 1:**
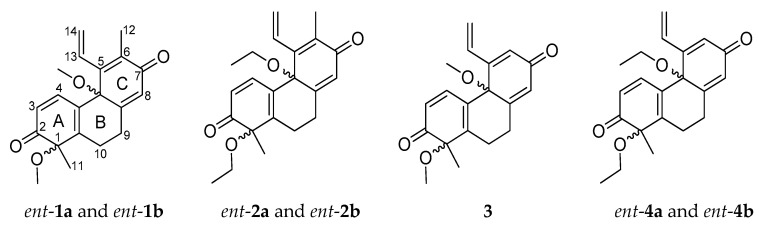
The structures of semisynthetic derivatives of juncusol (**1**,**2**) and effusol (**3**,**4**).

Four semisynthetic derivatives (**1**–**4**) were prepared from juncusol and effusol, in four transformations (I–IV), using a hypervalent iodine(III) reagent in a diversity-oriented approach (Scheme 1 and Figure 1.). While many oxidants are available for the oxidation of phenols, the use of [bis-(trifluoroacetoxy)]iodobenzene (PIFA) was a natural follow-up to our previous work on the diversity-oriented transformation of phenolic natural products. This reagent allows selective transformation of phenolic hydroxyl groups under mild conditions, and we have previously found it to be efficient in the (i) synthesis of functionalized quinol derivatives with antitumor potential [27,37], and (ii) simulation of certain biomimetic oxidative conditions [38]. The latter is because it may act not only through an aryloxyiodonium(III) intermediate forming a phenoxenium ion that undergoes a nucleophilic attack, but it can also oxidize aromatic compounds via a single-electron transfer mechanism [39,40]. In this work, PIFA was used in MeCN–MeOH (processes I and III), or MeCN–EtOH (II and IV), at room temperature, and this led to the formation of quinol-type products. Since both abovementioned reaction mechanisms orient subsequent coupling reaction steps to the *ortho* or *para* positions, these products were expectably *o*- and/or *p*-quinol derivatives. Following the oxidation, the mixture of products was subjected to a solid-phase extraction on silica gel, to remove the remaining oxidizing agent and the oxidation side-products. The purification process was followed by MPLC and HPLC.

During the reaction processes, the mixture of products, bearing *o*- and *p*-quinol rings and substituted by methoxy- and ethoxy groups, were formed according to the solvent used. As the result of chromatographic purifications, all compounds were obtained as diastereomeric mixtures of two racemates.

The structure determination was carried out by extensive spectroscopic analysis, using HRESIMS, and 1D and 2D NMR (^1^H–^1^H COSY, HSQC, and HMBC) measurements. The structures of the semisynthetic derivatives are depicted in Figure 1.

**Figure 2:**
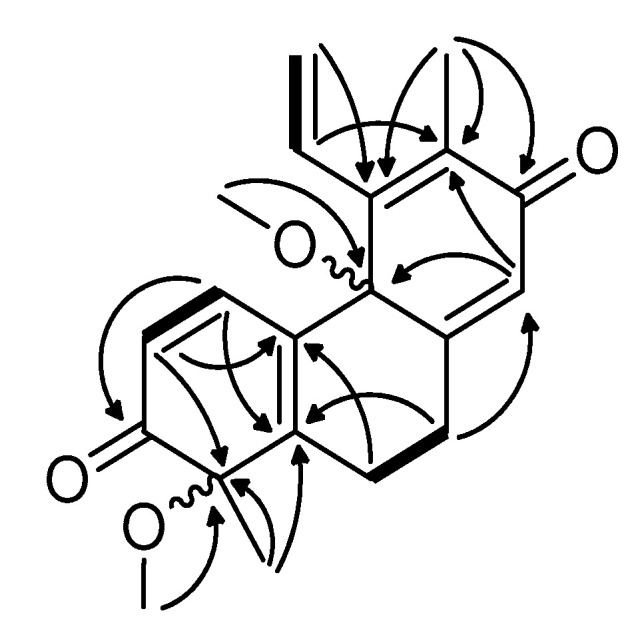
Diagnostic COSY (▬) and HMBC (H→C) correlations of compounds **1a** and **1b**.

The ^1^H-NMRspectrum of **1a** and **1b** showed that these compounds are racemic diastereomers (Figures S1 and S7). Since these compounds are derived from juncusol, similar signals were found in the ^1^H-NMR spectrum: resonances of two *o*-coupled aromatic protons (δ_H_ 5.96 dd/5.91 dd and 6.83 d/6.88 d), two methyls (δ_H_ 1.26 s/1.14 s and 2.15 s/2.16 s), a vinylic system at δ_H_ 6.89 dd/6.91 dd, 6.01 dd/6.02 dd, and 5.96 dd/5.69 dd (H-13, H_2_-14), two methylene groups (δ_H_ 2.89 m/2.91 m, 2.48 m/2.58 m and δ_H_ 3.00 m/2.95 m, 2.79 ddd/2.95 m), and signals of two methoxy groups (δ_H_ 3.15 s/2.92 s and 3.00 s/3.02 s) (Table 1). The presence of two methylene signals (H_2_-9 and H_2_-10) indicated this racemic pair to be 9,10-dihydrophenanthrene derivatives. In the JMOD (*J*-modulated spin‒echo experiment) spectrum, 20 carbon signals were displayed (Table 2). In the ^1^H‒^1^H COSY spectrum, correlations were observed between δ_H_ 5.96 dd/5.91 dd and δ_H_ 6.83 d/6.88 d (H-3–H-4), between δ_H_ 2.89 m/2.91 m, 2.48 m/2.58 m and δ_H_ 3.00 m/2.95 m, 2.79 ddd (H-9–H-10) and between δ_H_ 6.89 dd/6.91 dd and δ_H_ 6.01 dd/6.02 dd, 5.96 dd/5.69 dd (H-13–H_2_-14) (Figure 2.). The locations of the methoxy groups were concluded from the HMBC spectra, as the proton signal at δ_H_ 3.15/2.92 (CH_3_O-1) gave cross-peaks with δ_C_ 84.7/82.4 (C-1), whereas the signal at δ_H_ 3.00/3.02 (CH_3_O-5a) correlated with δ_C_ 77.5/76.8 (C-5a) (Figure 2.). Thus, the C-2 and C-7 hydroxy groups of juncusol were oxidized to carbonyl groups (δ_C_ 202.6/202.9 and 184.9/184.8, respectively), and methoxy-substituted dienone moieties were formed.

All of the above evidence confirmed the 2D structures of compounds **1a** and **1b**.

Compounds **2a** and **2b** were also obtained as racemic diastereomers, originated from juncusol. The difference between compounds **1a** and **1b** and compounds **2a** and **2b** involved the replacement of the C-1 and C-5a methoxy groups with ethoxy substituents (Table 1 and Table 2). Similar to **1a** and **1b**, differences were found between the ^13^C-NMRchemical shifts of C-1 and adjacent carbon C-1a in **2a** and **2b**, which indicated the opposite stereochemistry in chiral center C-1 in these compounds.

In the case of compound **3**, a derivative of effusol, the signals of two *o*-coupled aromatic protons (δ_H_ 6.01 dd and 6.98 d, H-3 and H-4), two aromatic protons as a singlet (6.62 s and 6.28 s; H-6 and H-8), one methyl (δ_H_ 1.27 s), a vinyl group (δ_H_ 6.64 dd, 6.08 dd, and 5.63 d, H-13, H_2_-14), two methylene groups (2.89 m, 2.55 m and δ_H_ 2.99 m, 2.77 ddd, H_2_-9 and H_2_-10), and two methoxy groups (δ_H_ 3.15 s and 3.06 s) were detected in the ^1^H-NMRspectrum (Table 1). The differences between effusol and compound **3** involved the oxidation of the phenolic hydroxy groups at C-2 and C-7 to carbonyl moieties (δ_c_ 202.3 and 184.9, respectively), and the addition of methoxy groups at C-1 and C-5a (Table 2). Although compound **3** could not be separated into racemic pairs like **2a** and **2b** or **4a** and **4b**, the presence of four stereoisomers can be supposed with regard to the similar reaction process.

Compounds **4a** and **4b** differ from compound **3** in the presence of ethoxy side chains (δ_H_ 3.18 m, 2.75 m (C*H*_2_), 1.16 t (C*H*_3_), and 3.24 m, 3.11 m (C*H*_2_), 1.16 t (C*H*_3_)) at C-1 and C-5a in case of **4a**, and (δ_H_ 3.38 m, 3.11 m (C*H*_2_), 1.24 t (C*H*_3_), and 3.24 m, 3.11 m (C*H*_2_), 1.15 t (C*H*_3_)) at the same positions in **4b**, as it was deduced from the relevant HMBC interactions. Similar to compound **3**, oxidation at C-2 (δ_C_ 202.2) and C-7 (δ_C_ 185.1) to carbonyl groups was observed. The main difference between **4a** and **4b**, detected in the ^13^C-NMRspectra, was the distinct chemical shifts of C-1 (**4a**: δ_C_ 81.8 and **4b**: δ_C_ 84.0) and C-1a (**4a**: δ_C_ 160.5 and **4b**: δ_C_ 153.6), suggesting that the two compounds have the opposite stereochemistry in chiral center C-1.

### 2.2. Antiproliferative Activity of the Isolated Phenanthrenes

The antiproliferative effects of the isolated natural phenanthrenes and the semisynthetic products (**1b**, **2a**, **2b**, **3**, **4a**, and **4b**) were investigated by a standard MTT method on human breast (MCF-7, KCR, T47D, and HTB-26), cervical (HeLa), and ovarian (A2780 and A2780cis) cancer cells, and on MRC-5 (human embryonic lung fibroblast) cell lines. Previously, juncusol and effusol were described as having promising antiproliferative activity (IC_50_ values 0.95 μM for juncusol, and 3.68 μM for effusol on HeLa cells) [7,41]. Juncusol was found to be nontoxic on normal skin fibroblasts CCD966SK, even at higher concentrations, but increased the number of cells in the G2/M and subG1 cell cycle phase even at lower concentrations. Moreover, juncusol caused a significant and concentration-dependent increase in caspase-3 activity, and also decreased tubulin polymerization at 200 µM [41].

In our investigations, among the natural phenanthrenes, 2,7-dihydroxy-1,8-dimethyl-5-vinyl-9,10-dihydrophenanthrene was the most active on all tested cell lines, with the exception of HeLa (Table 3). The only difference between juncusol and effusol is the presence of a methyl group at C-6 in juncusol. Juncusol and effusol possessed significant antiproliferative activity on HeLa cells (IC_50_ values 0.5 μM for juncusol, and 2.3 μM for effusol, respectively). Compound **3** was found to be the most promising semisynthetic component with substantial antiproliferative effects against all tested cell lines, except for KCR, which was comparable to that of the positive control, cisplatin. Unfortunately, **3** had antiproliferative activity (IC_50_ = 12.2 μM) against the non-tumoral MRC-5 cells. This represents a ca. 2.1-fold selectivity against MCF-7 cells, which greatly over-performs the anticancer drug cisplatin that showed an opposite selectivity and was 2.3-fold more cytotoxic on MRC-5 cells (Table 3). Compounds **4a** and **4b** showed marked antiproliferative activity against MCF-7 cells (IC_50_ values 11.7 μM for **4a** and 10.2 μM for **4b**, respectively). In the case of 2,7-dihydroxy-1,8-dimethyl-5-vinyl-9,10-dihydrophenanthrene, IC_50_ value 12.9 μM was detected on MCF-7 cells. In case of effusol derivatives, the semisynthetic compounds had higher antiproliferative activities than that of the parent compound on all cell lines except on HeLa. None of the juncusol derivatives exceeded the antiproliferative effects of the parent compound. Although compounds **1b** and **2a** did not show an antiproliferative effect against normal (MRC-5) cells at tested concentrations, they possessed very weak activity against the investigated tumor cell lines. Comparing the data of effusol and juncusol derivatives, it can be stated that the presence of a methyl group at C-6 in the semisynthetic compounds resulted in a decrease of the toxic effect.

Juncuenin B, a natural phenanthrene isolated from *Juncus inflexus*, differs from juncusol in only the position of substituents (hydroxy at C-6, methyl at C-7, and vinyl at C-8) on ring C. In our previous investigation, semisynthetic derivatives of juncuenin B were prepared [27]. Some of these compounds contain the same structural elements (*o*- and *p*-quinoidal structure; methoxy- and ethoxy-substitution) as juncusol derivatives produced in this experiment. In that case, one of the joining methoxy or ethoxy groups connects at C-8a instead of C-5a. In the antiproliferative assay, these compounds showed higher activity; therefore, besides the connecting position of alkyl chains, the position of hydroxy, methoxy, and vinyl groups on ring C also has an important effect on the antiproliferative activity.

## 3. Materials and Methods

### 3.1. General

NMR spectra were recorded in methanol-*d*_4_ and CDCl_3_, on a Bruker Avance DRX 500 spectrometer (Bruker Biospin GmbH, Rheinstetten, Germany), at 500 MHz (^1^H) and 125 MHz (^13^C). The signals of the deuterated solvents were taken as references. The chemical shift values (δ) are given in ppm, and coupling constants are in Hz. Two-dimensional experiments were performed with standard Bruker software. In the ^1^H–^1^H COSY, HSQC, and HMBC experiments, gradient-enhanced versions were applied. The HRMS spectra were acquired on a Thermo Scientific Q-Exactive Plus Orbitrap mass spectrometer (Thermo Fisher Scientific Inc., Budapest, Hungary) equipped with ESI ion source in positive ionization mode. The data were acquired and processed with the MassLynx software (Waters Corporation, Budapest, Hungary).

In the reaction processes, [bis(trifluoroacetoxy)iodo]benzene (PIFA) was used as oxidizing agent (Sigma-Aldrich, Stockholm, Sweden). The progress of reactions and the efficacy of separation were monitored by TLC on Kieselgel 60F_254_ silica plates obtained from Merck (Merck, Darmstadt, Germany) and examined under UV light, at 254 and 366 nm. Solid phase extraction (SPE) was carried out on silica gel (Kieselgel 60 GF_254_, 15 µm, Merck). Flash chromatography was performed by a Combi Flash Rf^+^ Lumen instrument (Teledyne Isco). Normal phase vacuum liquid chromatography (VLC) was carried out on silica gel (Kieselgel 60 GF_254_, 15 µm, Merck). Rotation planar chromatography (RPC) was carried out by a Chromatotron instrument (Model 8924, Harrison Research, Palo Alto, CA, USA). All solvents used for MPLC, VLC, and RPC were of at least analytical grade (VWR Ltd., Szeged, Hungary). The isolated compounds, reaction mixtures, and processed fractions were investigated by HPLC, on a Waters instrument equipped with a Waters 2998 PDA detector, with an Agilent Eclipse XDB C-8 (9.4 × 250 mm, 5 µm) (Kromat Kft Budapest, Hungary) and Zorbax ODS (5 µm, 9.4 *×* 250 mm) columns. MeCN–H_2_O solvent system was used as eluent. The data were acquired and processed with the Empower software (Waters Corporation, Budapest, Hungary)

### 3.2. Plant Material

Aerial parts of *Juncus tenuis* Willd. were collected during the flowering period in the Botanical Garden of the University of Szeged, in June 2019. The plant material was identified by László Bakacsy (Department of Plant Biology, University of Szeged, 6726 Szeged, Hungary) and Dragica Purger (Department of Pharmacognosy, University of Pécs, 7624 Pécs, Hungary). A voucher specimen (No. 889) was deposited at the Herbarium of the Department of Pharmacognosy, University of Szeged, Szeged, Hungary.

### 3.3. Extraction and Isolation

The air-dried aerial parts of *Juncus tenuis* (1.68 kg) were ground and percolated with MeOH (50 L), at room temperature. The crude MeOH extract was concentrated under reduced pressure (130.0 g); the residue was dissolved in 50% MeOH and subjected to solvent–solvent partitioning with *n*-hexane (1.5 L) and CH_2_Cl_2_ (3 L), respectively. After evaporation, the CH_2_Cl_2_ phase (22.5 g) was chromatographed by VLC, on silica gel, with a gradient system of cyclohexane–EtOAc–MeOH (from 98:2:0 to 5:5:1 (1000 mL/eluent); volume of each fractions was 100 mL), to yield 3 major fractions (I–III).

Fraction I (650 mg) was separated by flash chromatography, on normal-phase silica gel with *n*-hexane–EtOAc gradient (linear gradient from 96:4 to 5:95) solvent system, to obtain 2 subfractions (the volume of the collected fractions was 25 mL). Subfraction 1/2 was re-chromatographed on reversed-phase flash chromatography, with H_2_O-MeOH gradient (linear gradient from 8:2 to 0:1) elution, and juncusol (176 mg) and effusol (207 mg) were obtained. Fraction III (270 mg) was further chromatographed by RPC, on silica gel, using *n*-hexane–acetone gradient system (from 9:1 to 0:1, volume of the collected fractions was 15 mL), to obtain 2 subfractions. Subfraction III/2 was purified by RP-HPLC, applying a Zorbax ODS (5 µm, 9.4 × 250 mm) column and MeOH–H_2_O gradient system (linear gradient from 25:75 to 94:6) as eluent (flow rate 3.0 mL/min), and 2,7-dihydroxy-1,8-dimethyl-5-vinyl-9,10-dihydrophenanthrene (*t*_R_ = 24.3 min, 4.5 mg) was isolated.

### 3.4. Synthesis and Purification Process

In each reaction process, 50 mg of starting material (juncusol in reaction processes I and II, and effusol in III and IV) was dissolved at a concentration of 1 mg/mL, in the corresponding solvent (MeCN-MeOH 9:1 in reaction processes I and III, and in MeCN-EtOH 9:1 in II and IV), and stirred with 2 equivalents of [bis-(trifluoroacetoxy)]iodobenzene (PIFA), for 30 min, at room temperature. After evaporating the solvent under reduced pressure, SPE on silica gel was applied to absorb the remaining oxidizing agent and the possibly decomposed compounds.

Reaction mixture I was fractionated by MPLC with *n*-hexane–EtOAc gradient solvent system, to obtain 2 fractions (I/1-2). From fraction I/2, compounds **1a** and **1b** (5.3 mg both) were isolated by using an Agilent Eclipse XDB C-8 (9.4 × 250 mm, 5 µm) column and MeCN–H_2_O (1:1) as mobile phase (flow rate 3 mL/min). Reaction mixture II was fractionated by MPLC with *n*-hexane–EtOAc gradient solvent system, to obtain 2 fractions (II/1-2). Fraction II/1 was further purified by RP-HPLC, to gain compounds **2a** and **2b** (*t*_R_ = 15.3 and 17.3 min, 1.6 mg both), using an Agilent Eclipse XDB C-8 (9.4 × 250 mm, 5 µm) column and MeCN–H_2_O (1:1) as mobile phase (flow rate 3 mL/min). Reaction mixture III was fractionated by MPLC with a gradient solvent system of *n*-hexane–EtOAc, to obtain two fractions (III/1-2). From fraction III/2, compound **3** (4.3 mg) was isolated. Finally, reaction mixture IV was fractionated by MPLC with an *n*-hexane–EtOAc gradient system, to afford 4 fractions (IV/1-4). From fraction IV/1 compounds **4a** and **4b** (1.4 mg both) were isolated by RP-HPLC, using an Agilent Eclipse XDB C-8 (9.4 × 250 mm, 5 µm) column and MeCN–H_2_O (6:4) as eluent (*t*_R_ = 23.0 and 25.3 min, respectively).

#### 3.4.1. Compound *ent*-**1a**

Light brown solid; ^1^H and ^13^C-NMRdata, see Table 1 and Table 2; HRESIMS *m*/*z* 349.1412 [M + Na]^+^ (calcd for C_20_H_22_O_4_Na, 349.1416).

#### 3.4.2. Compound *ent*-**1b**

Light brown solid; ^1^H and ^13^C-NMRdata, see Table 1 and Table 2; HRESIMS *m*/*z* 349.1412 [M + Na]^+^ (calcd for C_20_H_22_O_4_Na, 349.1416).

#### 3.4.3. Compound *ent*-**2a**

Pale yellow solid; ^1^H and ^13^C-NMR data, see Table 1 and Table 2; HRESIMS *m*/*z* 355.1900 [M + H]^+^ (calcd for C_22_H_26_O_4_, 355.1904).

#### 3.4.4. Compound *ent*-**2b**

Pale yellow solid; ^1^H and ^13^C-NMR data, see Table 1 and Table 2; HRESIMS *m*/*z* 355.1900 [M + H]^+^ (calcd for C_22_H_26_O_4_, 355.1904).

#### 3.4.5. Compound **3**

Pale yellow solid; ^1^H and ^13^C-NMR data, see Table 1 and Table 2; HRESIMS *m*/*z* 313.1434 [M + H]^+^ (calcd for C_19_H_20_O_4_, 313.1434).

#### 3.4.6. Compound *ent*-**4a**

Pale yellow solid, ^1^H and ^13^C-NMR data, see Table 1 and Table 2; HRESIMS *m*/*z* 341.1746 [M + H]^+^ (calcd for C_21_H_24_O_4_, 341.1747).

#### 3.4.7. Compound *ent*-**4b**

Pale yellow solid, ^1^H and ^13^C-NMR data, see Table 1 and Table 2; HRESIMS *m*/*z* 363.1568 [M + Na]^+^ (calcd for C_21_H_24_O_4_Na, 363.1572).

### 3.5. Antiproliferative Assay

#### 3.5.1. Cell Lines

Breast cancer cell line MCF-7 (ATCC^®^ HTB-22) and the drug-resistant subline of the human breast cancer MCF-7 (ECACC 86012803; KCR) were purchased from LGC Promochem (Teddington, UK). Both cell lines were cultured in Eagle’s Minimal Essential Medium (EMEM, containing 4.5 g/L glucose) supplemented with a non-essential amino acid mixture, a selection of vitamins, and 10% heat-inactivated fetal bovine serum. In every third passage, 0.56 µg/mL doxorubicin was added to the medium, in order to maintain the ABCB1 (P-glycoprotein) expression in KCR cells. A2780 human ovarian cancer cell line (ECACC, European Collection of Authentical Cell Culture, Sigma Cat.no. 93112519) was purchased from Merck KGaA (Darmstadt, Germany). The cisplatin-resistant human ovarian cancer cell line A2780cis (ECACC European Collection of Authentical Cell Culture, Sigma Cat.no. 93112517) was purchased from Merck KGaA (Darmstadt, Germany). The human ovarian cancer cell lines were cultured in RPMI 1640 medium supplemented with 10% heat-inactivated fetal bovine serum. The RPMI 1640 medium of the cisplatin resistant cell line A2780 was supplemented with 1 µM cisplatin. HeLa (ATCC^®^ CCL-2™) human cervix carcinoma cell line was purchased from LGC Promochem (Teddington, UK). The cells were cultured in Eagle’s Minimal Essential Medium (EMEM, containing 4.5 g/L glucose) supplemented with a non-essential amino acid mixture, a selection of vitamins, and 10% heat-inactivated fetal bovine serum. MRC-5 human embryonal lung fibroblast cell line (ATCC^®^ CCL-171) was purchased from LGC Promochem, Teddington, UK. The cell line was cultured in Eagle’s Minimal Essential Medium (EMEM, containing 4.5 g/L glucose) supplemented with a non-essential amino acid mixture, a selection of vitamins, and 10% heat-inactivated fetal bovine serum. HTB-26 breast adenocarcinoma cell line was purchased from LGC Promochem, Teddington, UK. The cell line was cultured in RPMI 1640 medium supplemented with 10% heat-inactivated fetal bovine serum. T-47D (ATCC^®^ HTB-133™) ductal carcinoma cell line was purchased from LGC Promochem, Teddington, UK. The cells were cultured in RPMI 1640 medium supplemented with 10% heat-inactivated fetal bovine serum, 2 mM l-glutamine, 1 mM Na-pyruvate, and 100 mM Hepes. All of the cells were incubated at 37 °C, in a 5% CO_2_ and 95% air atmosphere.

#### 3.5.2. Antiproliferative Assay

The antiproliferative effect of the compounds was determined on human breast (MCF-7, KCR, T47D, and HTB-26), cervical (HeLa), and ovarian (A2780 and A2780cis) cancer cells, and on MRC-5 (human embryonic lung fibroblast) cell lines. The adherent cells were cultured in 96-well flat-bottomed microtiter plates, using EMEM supplemented with 10% heat-inactivated fetal bovine serum or RPMI 1640 supplemented with 10% heat-inactivated fetal bovine serum, respectively. The density of the cells was adjusted to 6 × 10^3^ cells in 100 μL per well, the cells were seeded for 24 h at 37 °C, with 5% CO_2_, and then the medium was removed from the plates, and fresh medium (100 μL per well) was added to the cells. The effects of increasing concentrations of compounds on cell proliferation were tested in 96-well flat-bottomed microtiter plates. The compounds were diluted in the appropriate medium; the dilutions of compounds were performed in separate plates and then added to the cells. The starting concentration of the compounds was 100 μM, and two-fold serial dilution was performed (concentration range: 100–0.19 μM). The culture plates were incubated at 37 °C for 72 h; at the end of the incubation period, 20 μL of MTT (thiazolyl blue tetrazolium bromide, Sigma) solution (from a stock solution of 5 mg/mL) was added to each well. After incubation at 37 °C for 4 h, 100 μL of sodium dodecyl sulfate (SDS) (Sigma) solution (10% in 0.01 M HCI) was added to each well, and the plates were further incubated, at 37 °C, overnight. Cell growth was determined by measuring the optical density (OD) at 540/630 nm, with a Multiscan EX ELISA reader (Thermo Labsystems, Cheshire, WA, USA). Mean IC_50_ values were obtained by best-fitting the dose-dependent inhibition curves in GraphPadPrism5 program (GraphPad Software version 5.00 for Windows, San Diego, CA, USA) from four parallel experiments for each cell line.

Inhibition of the cell growth was determined according to the formula below:

$$\text{Inhibition}\%=100-\left[\frac{\text{OD sample-OD medium control}}{\text{OD cell control-OD medium control}}\right]\times 100$$

Results are expressed in terms of IC_50_, defined as the inhibitory dose that reduces the proliferation of the cells exposed to the tested compounds by 50% [42].

## 4. Conclusions

From the methanolic extract of *J. tenuis*, three phenanthrenes, namely juncusol, effusol, and 2,7-dihydroxy-1,8-dimethyl-5-vinyl-9,10-dihydrophenanthrene, were isolated. All of them were isolated for the first time from the plant. Oxidation of juncusol and effusol with hypervalent iodine(III) reagent in CH_3_CN-alcohol media resulted in the identification of phenanthrene derivatives substituted with methoxy and ethoxy groups at C-1 and C-5a. The semisynthetic derivatives contain substituted *o*- and *p*-quinol rings. The semisynthetic derivatives are reported here for the first time. The substitution and position of the substituents on ring C significantly affect the antiproliferative activity of the compounds.

## Figures and Tables

**Table 1:** ^1^H-NMRspectroscopic data for compounds **1**–**4** (500 MHz, CDCl_3_, δ ppm, and *J* in Hz).

Position	1a	1b	2a	2b	3	4a	4b
3	5.96 dd (10.3, 1.1)	5.91 dd (10.3, 1.1)	5.89 d (10.2)	5.94 d (10.3)	6.01 dd (10.3, 0.9)	5. 93 d (10.3)	5.98 d (10.3)
4	6.83 d (10.3)	6.88 d (10.3)	6.84 d (10.2)	6. 80 d (10.3)	6.98 d (10.3)	6.96 d (10.3)	6.94 d (10.3)
8	6.29 s	6.29 br s	6.24 s	6.24 s	6.28 s	6.23 s	6.23 s
9	2.89 m, 2.48 m	2.91 m, 2.58 m	2.87 m, 2.51, m	2.87 m, 2.47 m	2.89 m, 2.55 m	2.57 m, 2.91 m	2.87 m, 2.53 m
10	3.00 m, 2.79 ddd (17.4, 8.3, 1.6)	2.95 m (2H)	2.99 m, 2.93 m	2.99 m, 2.82 m	2.99 m (2H)	2.99 m, 2.86 m	2.81 m, 2.47 m
11	1.26 s	1.14 s	1.42 s	1.25, s	1.27 s	1.42 s	1.27 s
12	2.15 s	2.16 s	2.14 s	2.14 s			
13	6. 89 dd (17.8, 10.7)	6.91 dd (17.8, 11.9)	6.90 dd (17.7, 11.9)	6.88 dd (17.7, 11.8)	6.64 dd (17.7, 11.3)	6.64 dd (17.7, 11.3)	6.64 dd (17.7, 11.3)
14	6.01 dd (17.8, 1.3) 5.96 dd (10.7, 1.1)	6.02 dd (17.8, 1.3) 5.69 dd (11.9, 1.3)	6.05 d (17.7) 5.67 d (11.9)	6.03 d (17.7) 5.67 d (11.8)	6.08 br d (17.7) 5.63 br d (11.3)	6.10 d (17.7) 5.62 d (11.3)	6.09 d (17.7) 5.62 d (11.3)

**1a**: CH_3_O-1: 3.15 s; CH_3_O-5a: 3.00 s; **1b**: CH_3_O-1: 2.92 s; CH_3_O-5a: 3.02 s; **2a**: C_2_H_5_O-1: 3.15 dq (14.1, 7.0), 2.73 dq (14.1, 7.0) (C*H*_2_), 1.16 t (7.0) (C*H*_3_), C_2_H_5_O-5a: 3.20 dq (14.3, 7.0), 3.04 dq (14.3, 7.0) (C*H*_2_), 1.12 t (7.0) (C*H_3_*); **2b**: C_2_H_5_O-1: 3.37 dq (14.2, 7.0), 3.13 dq (14.2, 7.0) (C*H*_2_), 1.24 t (7.0) (C*H*_3_); C_2_H_5_O-5a: 3.20 dq (14.1, 7.0), 3.04 dq (14.1, 7.0), 1.11 t (7.0), 6-CH_3_: 2.14 s; **3**: CH_3_O-1: 3.15 s; CH_3_O-5a: 3.06 s; **4a**: C_2_H_5_O-1: 3.18 m, 2.75 m (*CH*_2_), 1.16 t (7.0) (C*H*_3_); C_2_H_5_O-5a: 3.24 m, 3.11 m (C*H*_2_), 1.16 t (7.0) (C*H*_3_)*;***4b**: C_2_H_5_O-1: 3.38 m, 3.11 m (C*H*_2_), 1.24 t (7.0) (C*H*_3_); C_2_H_5_O-5a: 3.24 m, 3.11 m (C*H*_2_), 1.15 t (7.0) (C*H*_3_).

**Table 2:** ^13^C-NMRspectroscopic data for compounds **1**–**4** (125 MHz, CDCl_3_, and δ ppm).

9	1a	1b	2a	2b	3a	4a	4b
1	84.7, C	82.4, C	81.7, C	84.2, C	84.5, C	81.8, C	84.0, C
1a	157.6, C	160.4, C	160.7, C	158.1, C	157.7, C	160.5, C	153.6,C
2	202.6, C	202.9, C	203.1, C	202.8, C	202.3, C	202.4, C	202.5,C
3	123.7, CH	123.1, CH	123.0, CH	123.6, CH	124.4, CH	123.8, CH	124.3, CH
4	142.2, CH	142.2, CH	142.2, CH	142.1, CH	141.1, CH	141.1, CH	141.1, CH
4a	132.4, C	132.6, C	132.1, C	132.0, C	131.8, C	131.3, C	131.3, C
5a	77.5, C	76.8, C	76.4, C	76.8, C	76.6, C	75.9, C	76.4, C
5	146.1, C	145.9, C	146.6, C	146.7, C	152.7, C	153.2, C	153.3, C
6	138.4, C	138.8, C	138.1, C	137.8, C	130.5, C	130.3, CH	130.0, CH
7	184.6, C	184.8, C	184.8, C	184.9, C	184.9, C	185.1, C	202.5, C
8	126.2, CH	125.9, CH	125.5, CH	125.8, CH	126.8, CH	126.0, CH	126.3, CH
8a	160.8, C	160.9, C	161.6, C	161.5, C	161.5, C	162.3, C	162.2, C
9	25.9, CH_2_	25.5, CH_2_	25.8, CH_2_	26.2, CH_2_	26.3, CH_2_	26.2, CH_2_	26.5, CH_2_
10	24.1, CH_2_	23.2, CH_2_	23.2, CH_2_	24.2, CH_2_	25.0, CH_2_	24.0, CH_2_	24.9, CH_2_
11	25.4, CH_3_	26.4, CH_3_	26.4, CH_3_	25.5, CH_3_	25.8, CH_3_	26.5, CH_3_	25.9, CH_3_
12	11.9, CH_3_	11.9, CH_3_	11.8, CH_3_	11.8, CH_3_			
13	132.1, CH	132.1, CH	132.2, CH	132.3, CH	134.4, CH	134.5, CH	134.5, CH
14	125.6, CH_2_	125.4, CH_2_	125.3, CH_2_	125.6, CH_2_	123.1, CH_2_	123.1, CH_2_	123.0, CH_2_

**1a**: CH_3_O-1: 55.0; CH_3_O-5a: 52.8; 1b: CH_3_O-1: 54.4; CH_3_O-5a: 52.9; **2a**: C_2_H_5_O-1: 62.3 (C*H*_2_), 15.0 (C*H*_3_); C_2_H_5_O-5a: 60.7 (*CH*_2_), 15.0 (C*H*_3_); **2b**: C_2_H_5_O-1: 63.0 (C*H*_2_), 15.8 (C*H*_3_), C_2_H_5_O-5a: 60.7 (C*H*_2_), 15.1 (C*H*_3_); **3**: CH_3_O-1: 55.0 (C*H*_3_), CH_3_O-5a: 52.9 (*CH*_3_); **4a**: C_2_H_5_O-1: 62.4 (C*H*_2_), 15.7 (C*H*_3_), C_2_H_5_O-5a: 61.0 (C*H*_2_), 15.2 (C*H*_3_); **4b**: C_2_H_5_O-1: 63.0 (*CH*_2_), 15.8 (C*H*_3_), C_2_H_5_O-5a: 61.0 (C*H*_2_), 15.2 (C*H*_3_).

**Table 3:** IC_50_ values of the compounds.

Compound	IC_50_ (µM) ± SD							
	A2780	A2780cis	KCR	MCF-7	HeLa	HTB-26	T47D	MRC-5
2,7-dihydroxy-1,8-dimethyl-5-vinyl-9,10-dihydrophenanthrene	22.3 ± 2.7	16.9 ± 4.7	24.2 ± 2.1	12.9 ± 0.2	24.7 ± 0.3	22.8 ± 0.2	14.2 ± 1.1	18.9 ± 4.0
juncusol	23.8 ± 1.3	37.1 ± 2.8	35.8 ± 1.7	37.1 ± 1.1	0.5 ± 0.0	41.7 ± 3.5	25.0 ± 0.4	40.9 ± 2.0
effusol	33.1 ± 3.1	30.4 ± 0.4	39.3 ± 1.6	48.6 ± 3.4	2.3 ± 0.7	57.0 ± 2.7	24.6 ± 1.9	60.1 ± 5.1
**1b**	80.3 ± 3.0	88.2 ± 3.1	>100	52.1 ± 4.8	>100	74.3 ± 3.6	36.5 ± 1.0	>100
**2a**	66.0 ± 4.4	62.9 ± 1.7	>100	80.5 ± 3.5	94.7 ± 3.4	94.3 ± 2.0	56.3 ± 3.4	>100
**2b**	39.4 ± 3.1	38.0 ± 4.6	44.2 ± 2.6	41.0 ± 0.9	61.9 ± 0.3	45.8 ± 3.2	30.3 ± 2.5	57.7 ± 0.3
**3**	8.6 ± 0.5	10.9 ± 2.2	18.9 ± 1.4	5.8 ± 0.2	12.9 ± 0.4	10.9 ± 0.9	7.0 ± 1.0	12.2 ± 0.2
**4a**	25.2 ± 1.8	22.5 ± 0.2	23.5 ± 0.8	11.7 ± 0.7	24.4 ± 0.8	16.1 ± 0.2	11.6 ± 0.3	14.3 ± 0.5
**4b**	22.0 ± 2.0	22.1 ± 1.6	29.4 ± 0.9	10.2 ± 0.1	35.0 ± 1.5	20.1 ± 1.1	14.2 ± 0.6	23.4 ± 1.3
Cisplatin	3.6 ± 0.3	7.3 ± 0.2	6.7 ± 0.4	1.4 ± 1.1	2.3 ± 0.1	20.1 ± 2.3	5.9 ± 0.1	0.6 ± 0.0
DMSO	>1	>1	>1	0.8 ± 0.1	>1	>1	0.8 ± 0.1	>1

Determinations were performed by MTT assay, by treating the cells with compounds (100–0.19 μM) for 72 h. Data are based on two independent experiments.

## Supplementary Materials

Supplementary materials are available online, Figures S1–S42.
