# Supplementary material for: Antiproliferative Phenanthrenes from Juncus tenuis: Isolation and Diversity-Oriented Semisynthetic Modification
Source: Molecules. 2020 Dec 17;25(24):5983. doi: 10.3390/molecules25245983 (PMC7765930; doi:10.3390/molecules25245983)
Supplement: Supplementary file 1 [file molecules-25-05983-s001.pdf]

Supporting Information

Antiproliferative Phenanthrenes from *Juncus tenuis*: Isolation and Diversity-Oriented Semisynthetic Modification

Csaba Bús [1], Norbert Kúsz [1], Annamária Kincses [2], Nikoletta Szemerédi [2], Gabriella Spengler [2], László Bakacsy [3], Dragica Purger [4], Róbert Berkecz [5], Judit Hohmann [1,6], Attila Hunyadi [1,*] and Andrea Vasas [1,*]

[1] Department of Pharmacognosy, University of Szeged, 6720 Szeged, Hungary; bcsaba0312@gmail.com (C.B.); kusznorbert@gmail.com (N.K.); hohmann.judit@szte.hu (J.H.)

[2] Department of Medical Microbiology and Immunobiology, University of Szeged, Dóm tér 10, 6720 Szeged, Hungary; kincses.annamaria90@gmail.com (A.K.); szemeredi.nikoletta@med.u-szeged.hu (N.S.); spengler.gabriella@med.u-szeged.hu (G.S.)

[3] Department of Plant Biology, University of Szeged, Közép Fasor 52, 6726 Szeged, Hungary; bakacsy@gmail.com

[4] Department of Pharmacognosy, University of Pécs, Rókus u. 2, 7624 Pécs, Hungary; dragica@gamma.ttk.pte.hu

[5] Institute of Pharmaceutical Analysis, University of Szeged, Somogyi u. 4, 6720 Szeged, Hungary; berkecz.robert@pharm.u-szeged.hu

[6] Interdisciplinary Centre of Natural Products, University of Szeged, Eötvös u. 6, 6720 Szeged, Hungary

* Correspondence: vasasa@pharmacognosy.hu (A.H.); hunyadi.a@pharmacognosy.hu (A.V.); Tel.: +36-62-546-451

Academic Editor: Piotr Paweł Wieczorek
Received: 23 November 2020; Accepted: 16 December 2020; Published: 17 December 2020

TABLE OF CONTENTS

Figure S1. ^{1}H NMR spectrum of compound **1a** (500 MHz, in CDCl3). 3

Figure S2. ^{13}C JMOD NMR spectrum of compound **1a** (125 MHz, in $CDCl_3$). 3

Figure S3. HSQC spectrum of compound **1a** (in $CDCl_3$). 4

Figure S4. ^{1}H-^{1}H COSY spectrum of compound **1a** (in $CDCl_3$). 4

Figure S5. HMBC spectrum of compound **1a** (in $CDCl_3$). 5

Figure S6. NOESY spectrum of compound **1a** (in $CDCl_3$). 5

Figure S7. ^{1}H NMR spectrum of compound **1b** (500 MHz, in $CDCl_3$). 6

Figure S8. ^{13}C JMOD NMR spectrum of compound **1b** (125 MHz, in $CDCl_3$). 6

Figure S9. HSQC spectrum of compound **1b** (in CDCl3). 7

Figure S10. ^{1}H-^{1}H COSY spectrum of compound **1b** (in $CDCl_3$). 7

Figure S11. HMBC spectrum of compound **1b** (in $CDCl_3$). 8

Figure S12. NOESY spectrum of compound **1b** (in $CDCl_3$). 8

Figure S13. ^{1}H NMR spectrum of compound **2a** (500 MHz, in $CDCl_3$). 9

Figure S14. ^{13}C JMOD NMR spectrum of compound **2a** (125 MHz, in $CDCl_3$). 9

Figure S15. HSQC spectrum of compound **2a** (in $CDCl_3$). 10

Figure S16. ^{1}H-^{1}H COSY spectrum of compound **2a** (in $CDCl_3$). 10

Figure S17. HMBC spectrum of compound **2a** (in $CDCl_3$). 11

Figure S18. NOESY spectrum of compound **2a** (in $CDCl_3$). 11
Figure S19. ^{1}H NMR spectrum of compound **2b** (500 MHz, in $CDCl_3$). 12
Figure S20. ^{13}C JMOD NMR spectrum of compound **2b** (125 MHz, in $CDCl_3$). 12
Figure S21. HSQC spectrum of compound **2b** (in $CDCl_3$). 13
Figure S22. ^{1}H-^{1}H COSY spectrum of compound **2b** (in $CDCl_3$). 13
Figure S23. HMBC spectrum of compound **2b** (in $CDCl_3$). 14
Figure S24. NOESY spectrum of compound **2b** (in $CDCl_3$). 14
Figure S25. ^{1}H NMR spectrum of compound **3** (500 MHz, in $CDCl_3$). 15
Figure S26. ^{13}C JMOD NMR spectrum of compound **3** (125 MHz, in $CDCl_3$). 15
Figure S27. HSQC spectrum of compound **3** (in $CDCl_3$). 16
Figure S28. ^{1}H-^{1}H COSY spectrum of compound **3** (in $CDCl_3$). 16
Figure S29. HMBC spectrum of compound **3** (in $CDCl_3$). 17
Figure S30. NOESY spectrum of compound **3** (in $CDCl_3$). 17
Figure S31. ^{1}H NMR spectrum of compound **4a** (500 MHz, in $CDCl_3$). 18
Figure S32. ^{13}C JMOD NMR spectrum of compound **4a** (125 MHz, in $CDCl_3$). 18
Figure S33. HSQC spectrum of compound **4a** (in $CDCl_3$). 19
Figure S34. ^{1}H-^{1}H COSY spectrum of compound **4a** (in $CDCl_3$). 19
Figure S35. HMBC spectrum of compound **4a** (in $CDCl_3$). 20
Figure S36. NOESY spectrum of compound **4a** (in $CDCl_3$). 20
Figure S37. ^{1}H NMR spectrum of compound **4b** (500 MHz, in $CDCl_3$). 21
Figure S38. ^{13}C JMOD NMR spectrum of compound **4b** (in $CDCl_3$). 21
Figure S39. HSQC spectrum of compound **4b** (in $CDCl_3$). 22
Figure S40. ^{1}H-^{1}H COSY spectrum of compound **4b** (in $CDCl_3$). 22
Figure S41. HMBC spectrum of compound **4b** (in $CDCl_3$). 23
Figure S42. NOESY spectrum of compound **4b** (in $CDCl_3$). 23

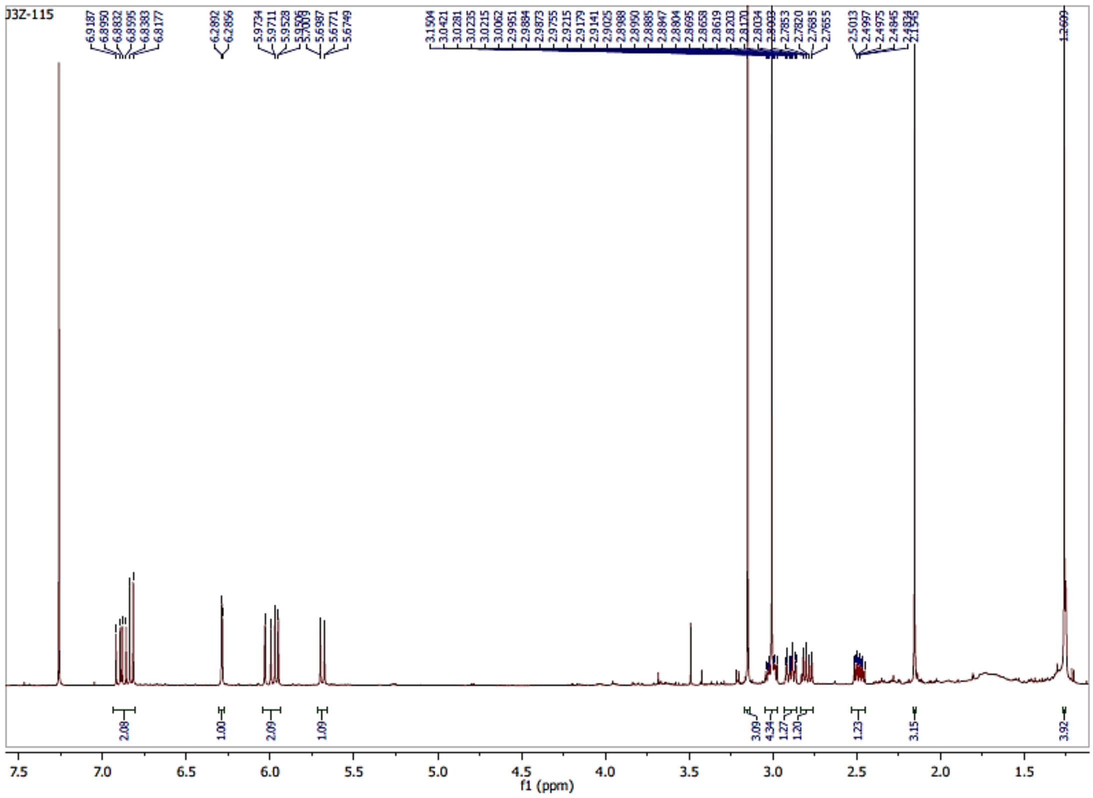

Figure S1. ^{1}H NMR spectrum of compound **1a** (500 MHz, in $CDCl_3$).

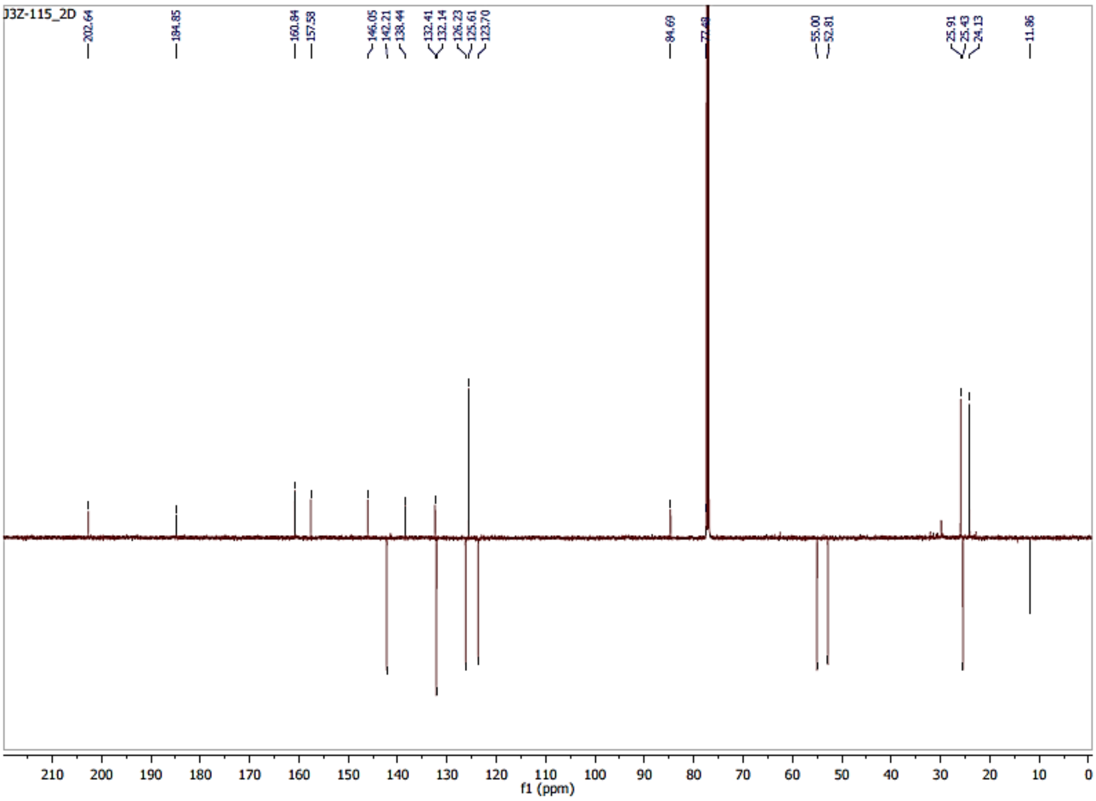

Figure S2. ^{13}C JMOD NMR spectrum of compound **1a** (125 MHz, in $CDCl_3$).

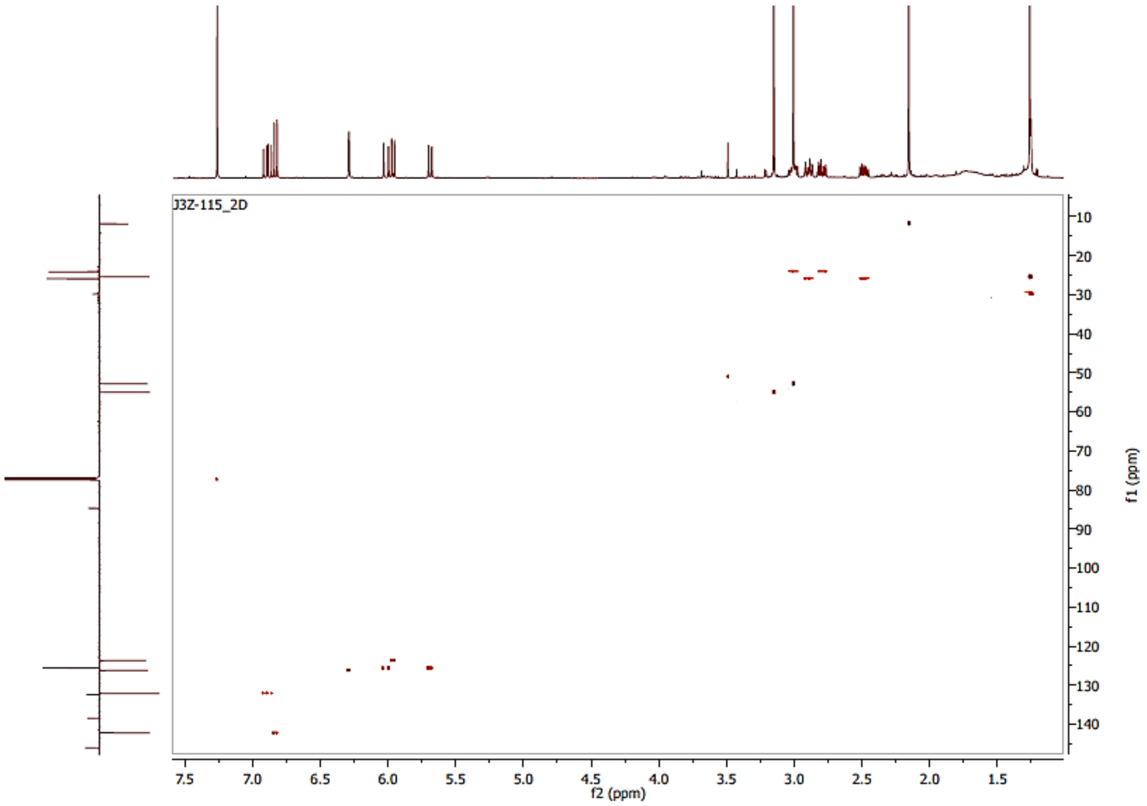

Figure S3. HSQC spectrum of compound **1a** (in $CDCl_3$).

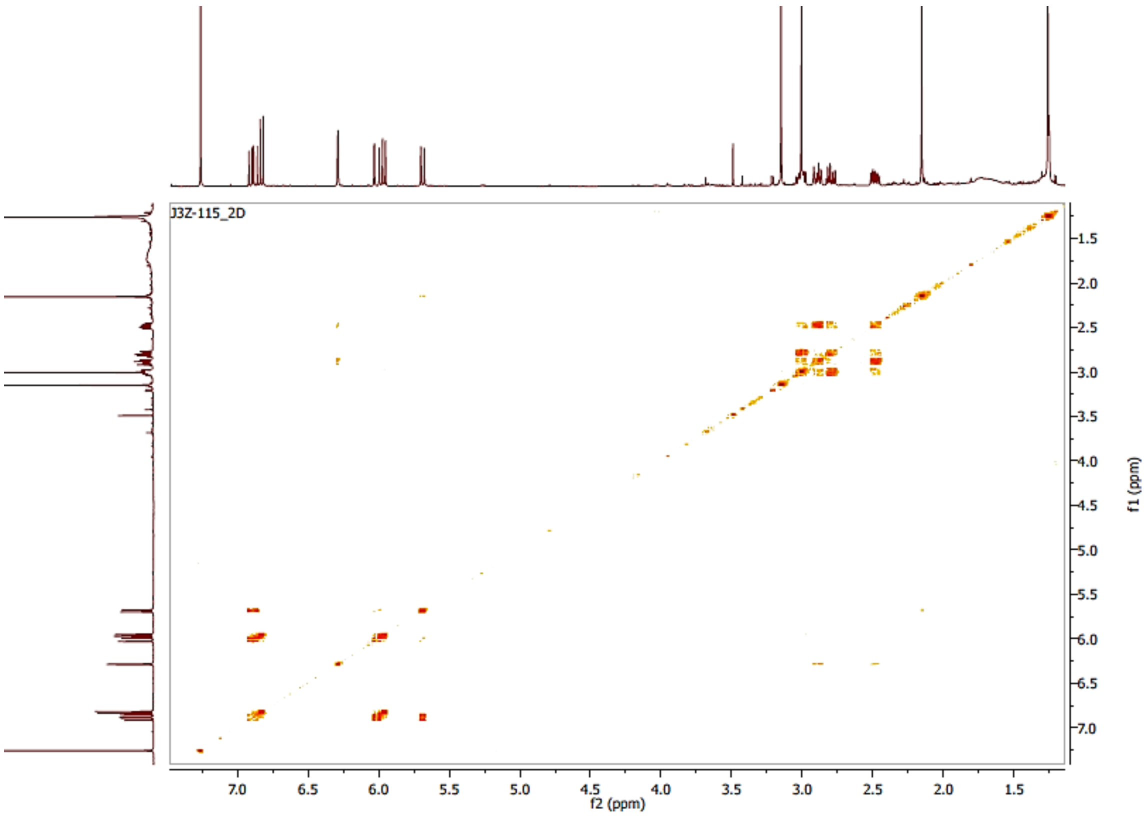

Figure S4. ^1H-^1H COSY spectrum of compound **1a** (in $CDCl_3$).

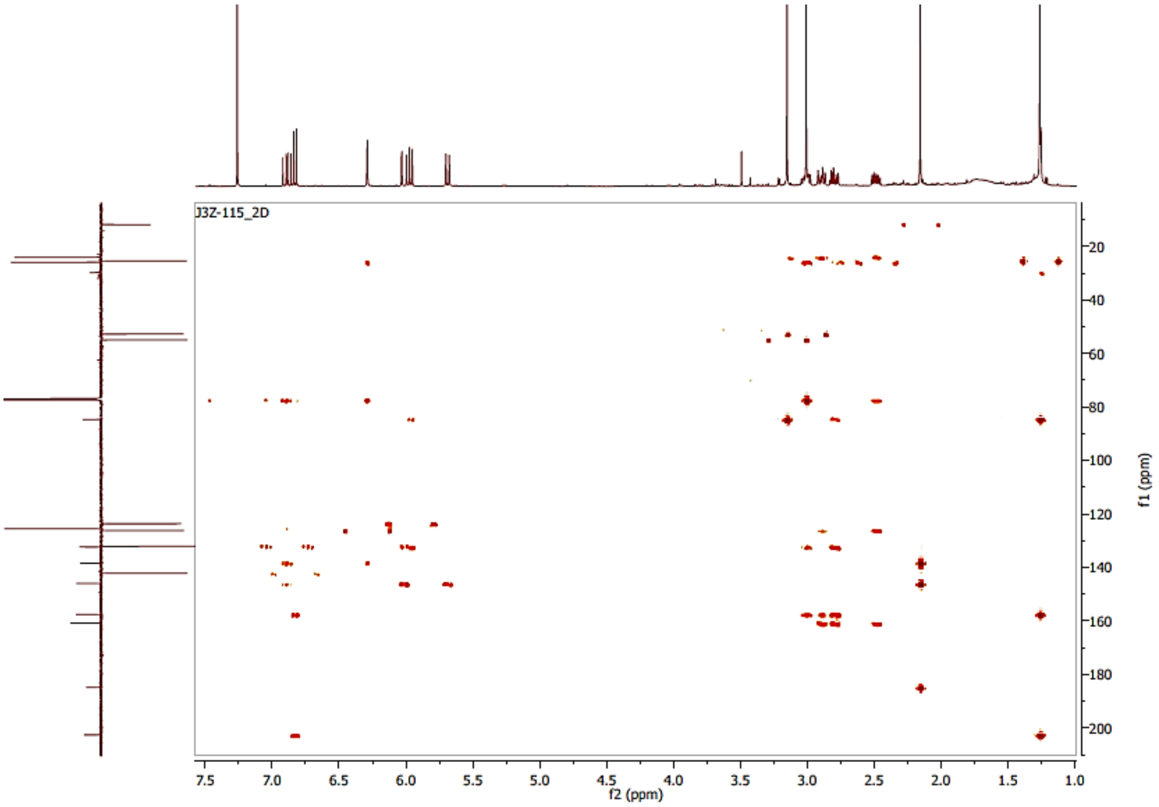

Figure S5. HMBC spectrum of compound **1a** (in $CDCl_3$).

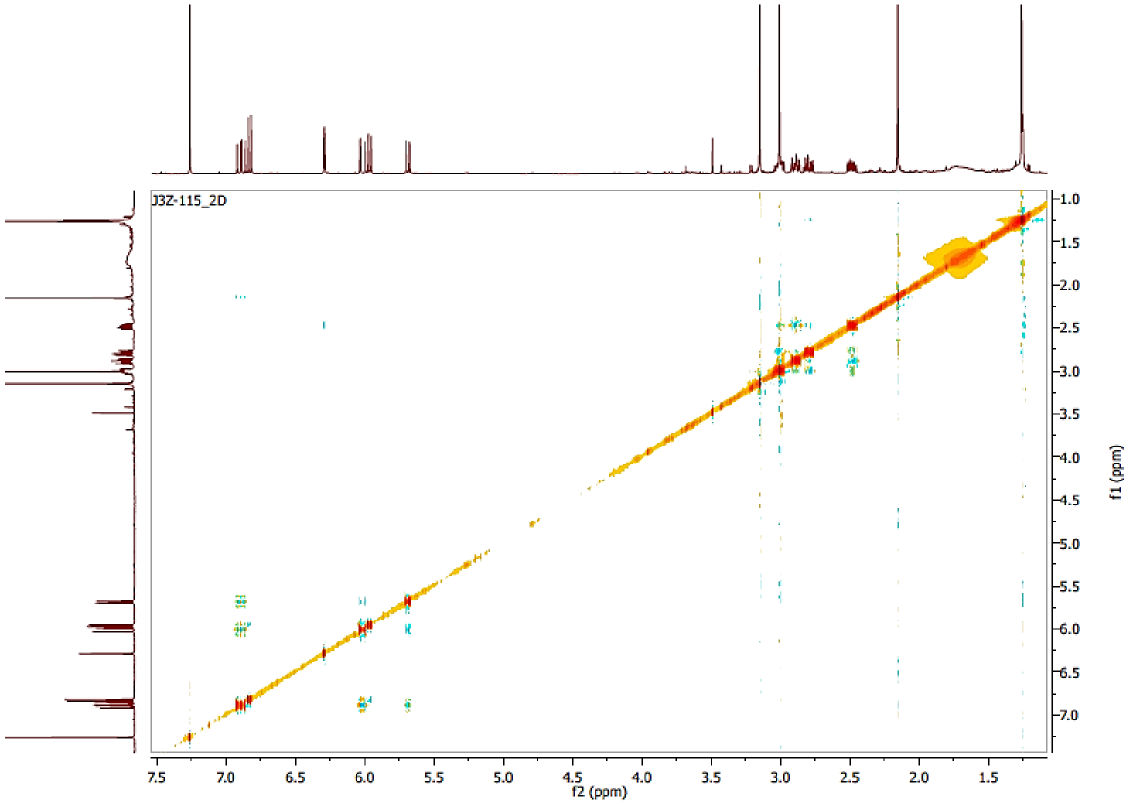

Figure S6. NOESY spectrum of compound **1a** (in $CDCl_3$).

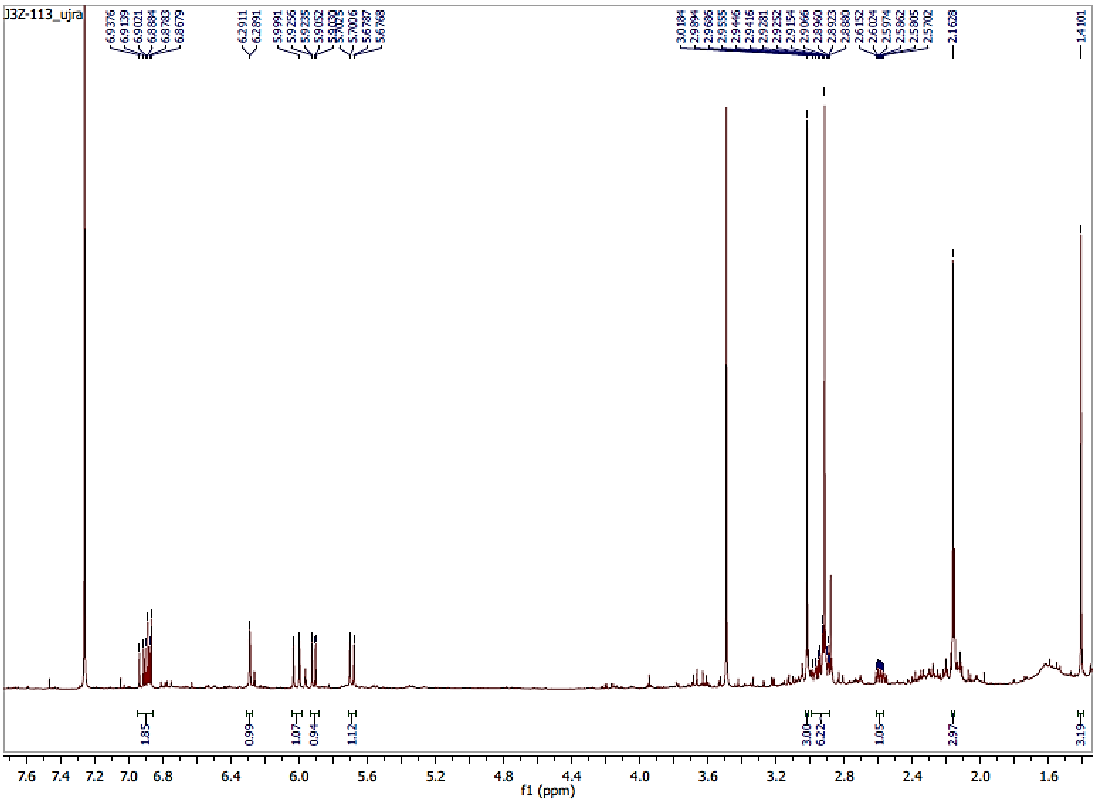

Figure S7. ^{1}H NMR spectrum of compound **1b** (500 MHz, in $CDCl_3$).

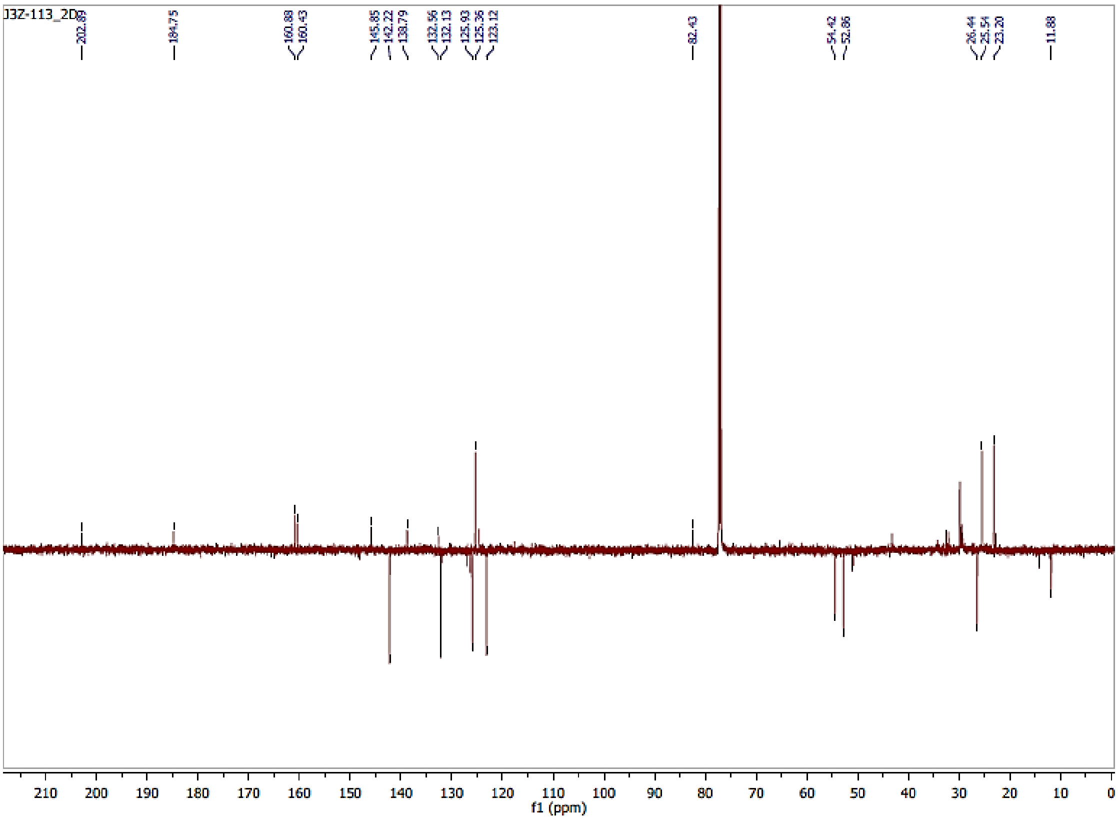

Figure S8. ^{13}C JMOD NMR spectrum of compound **1b** (125 MHz, in $CDCl_3$).

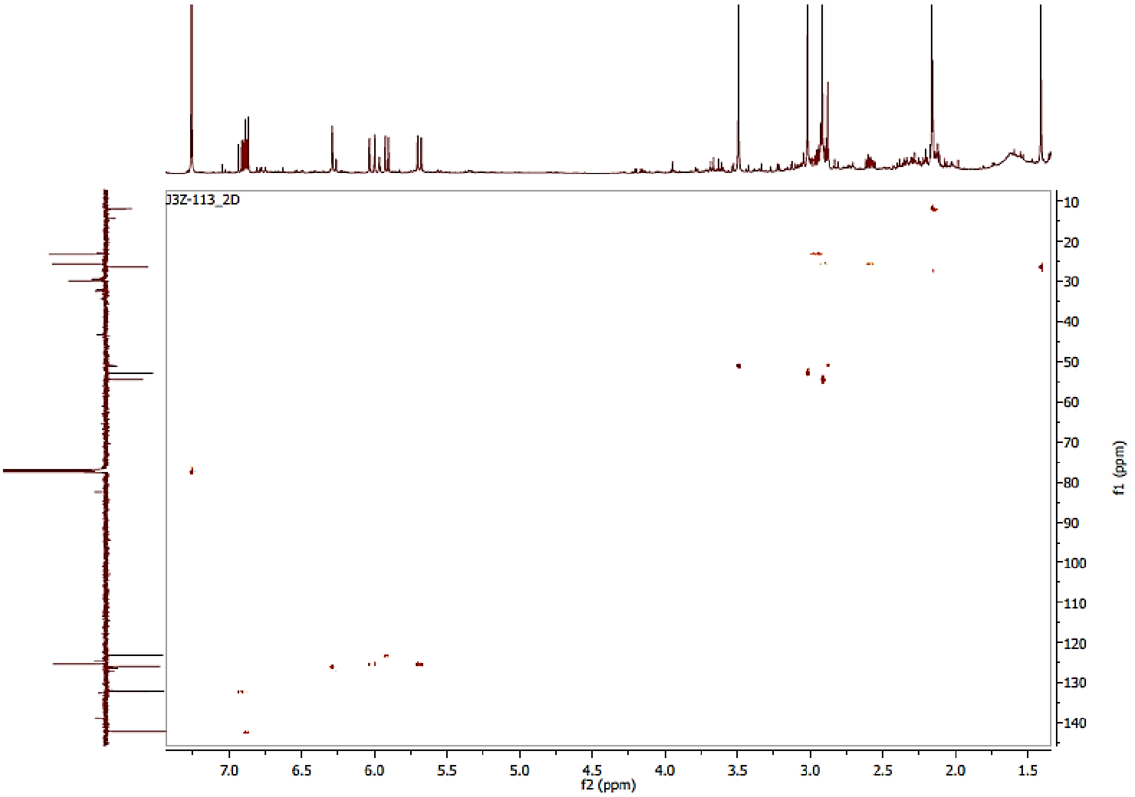

Figure S9. HSQC spectrum of compound **1b** (in CDCl3).

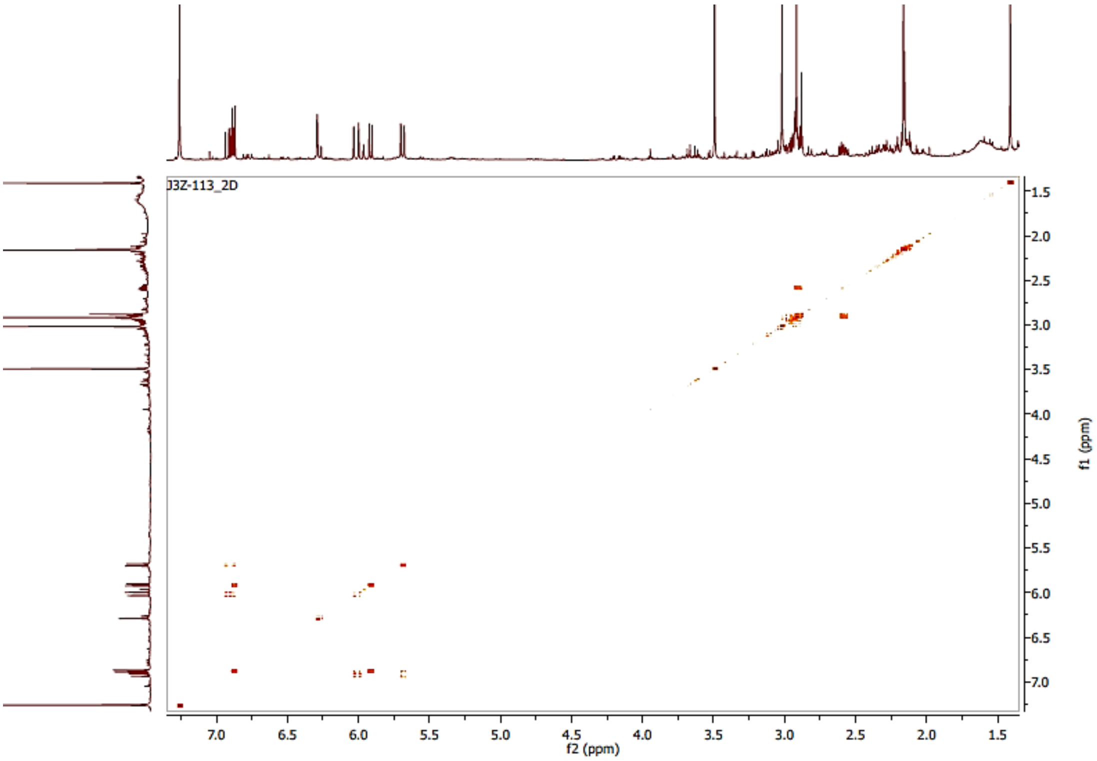

Figure S10. ^{1}H-^{1}H COSY spectrum of compound **1b** (in $CDCl_3$).

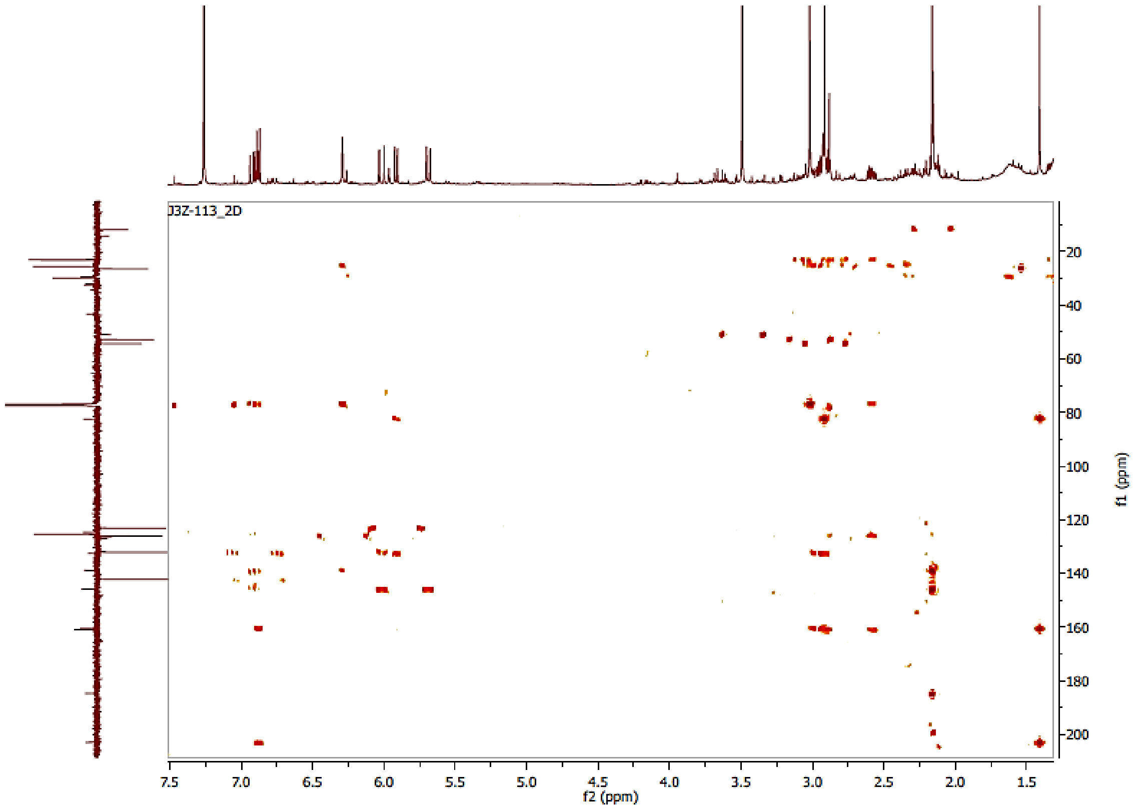

Figure S11. HMBC spectrum of compound **1b** (in $CDCl_3$).

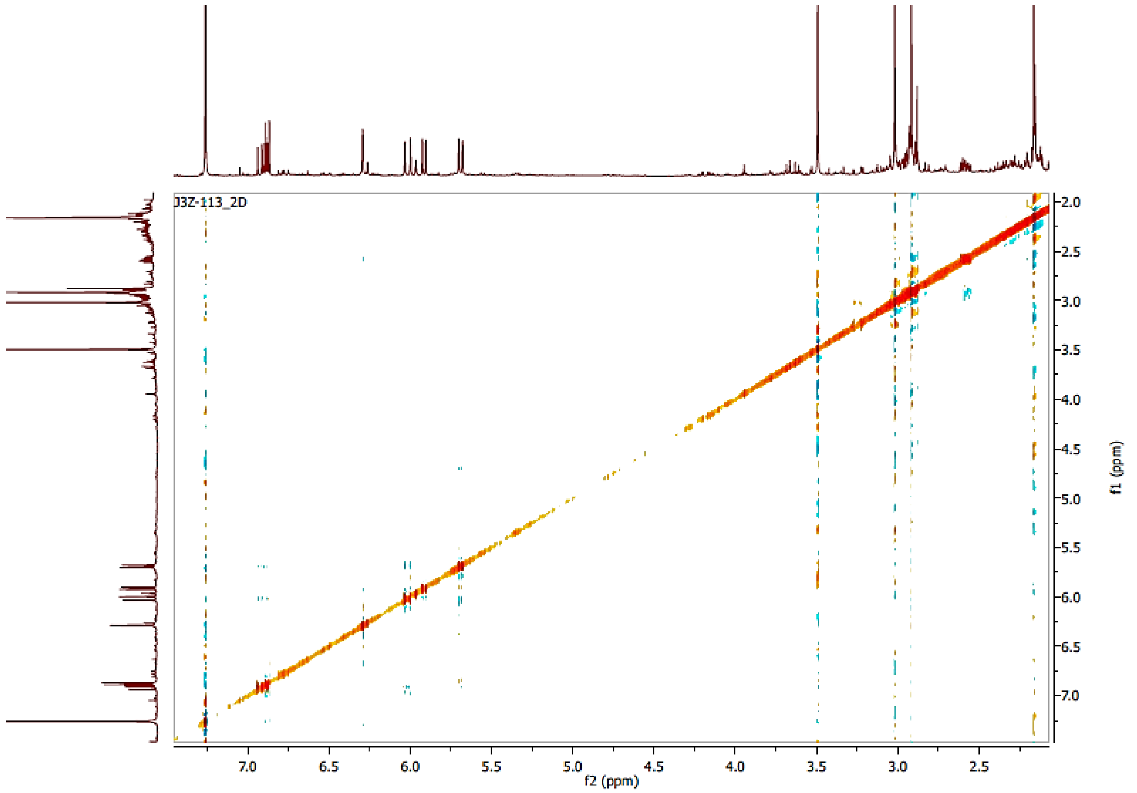

Figure S12. NOESY spectrum of compound **1b** (in $CDCl_3$).

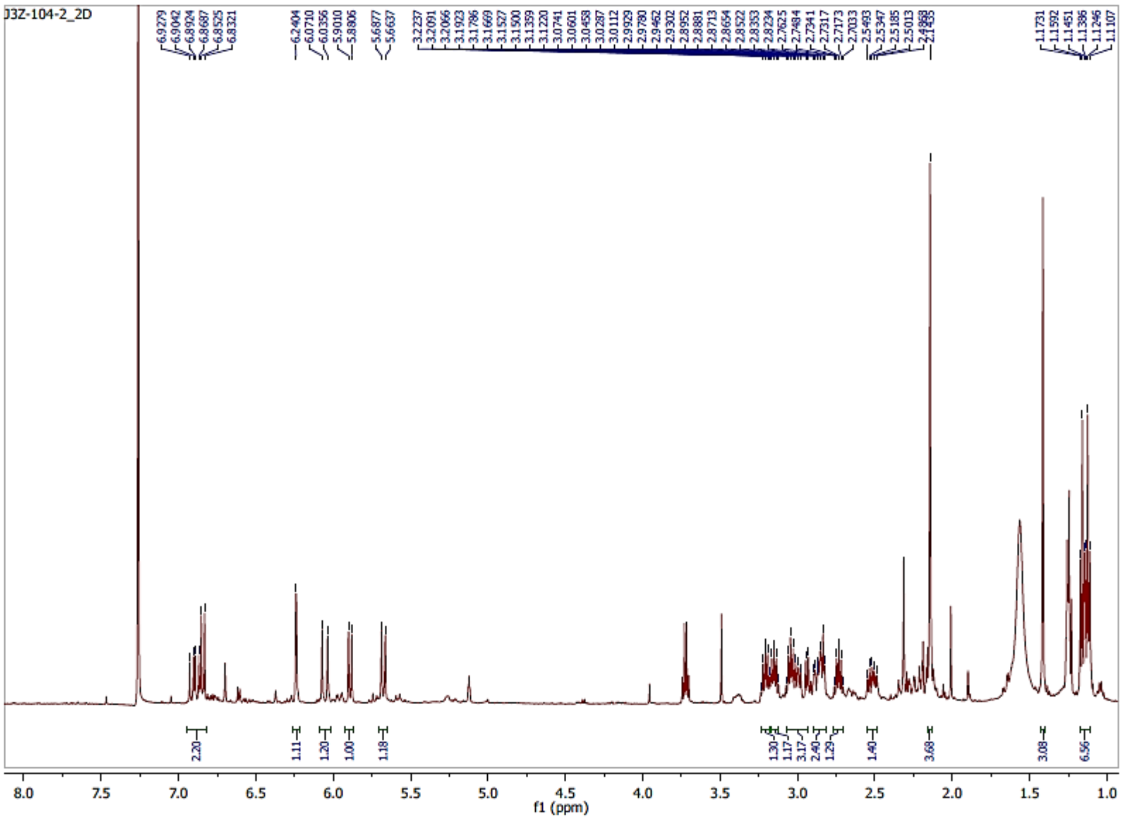

Figure S13. ^{1}H NMR spectrum of compound **2a** (500 MHz, in $CDCl_3$).

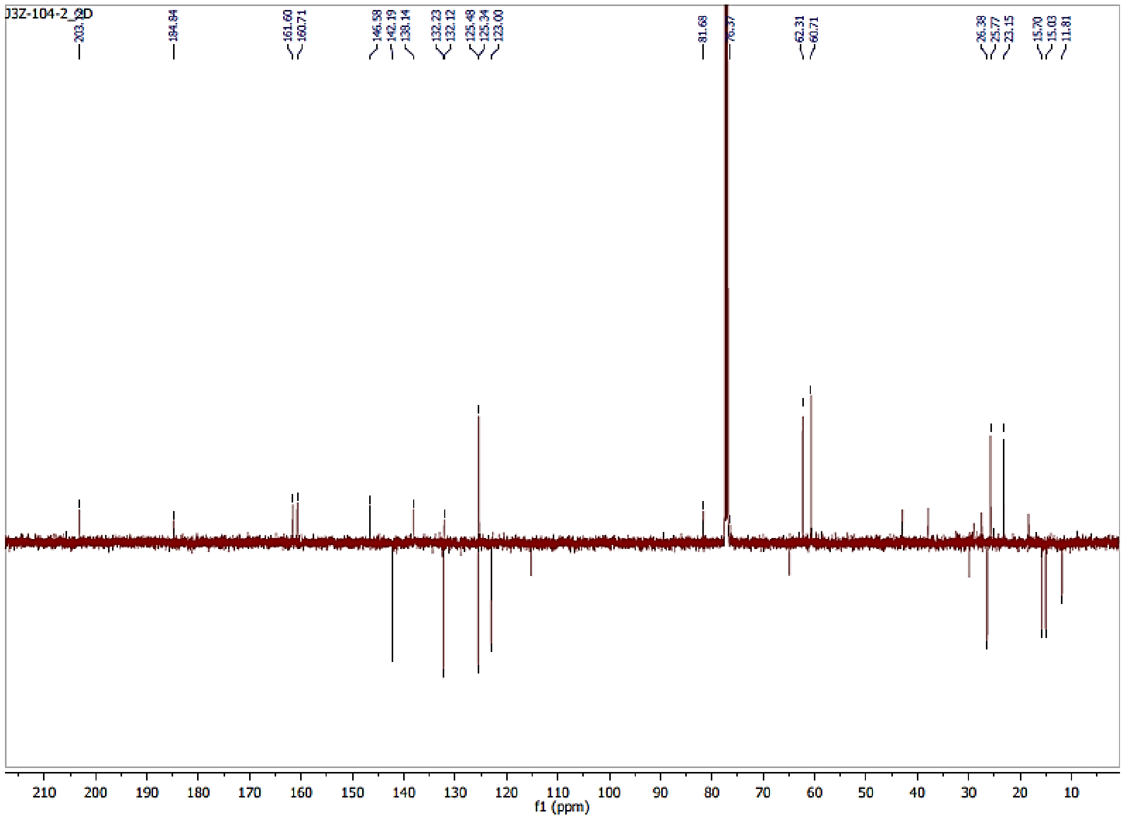

Figure S14. ^{13}C JMOD NMR spectrum of compound **2a** (125 MHz, in $CDCl_3$).

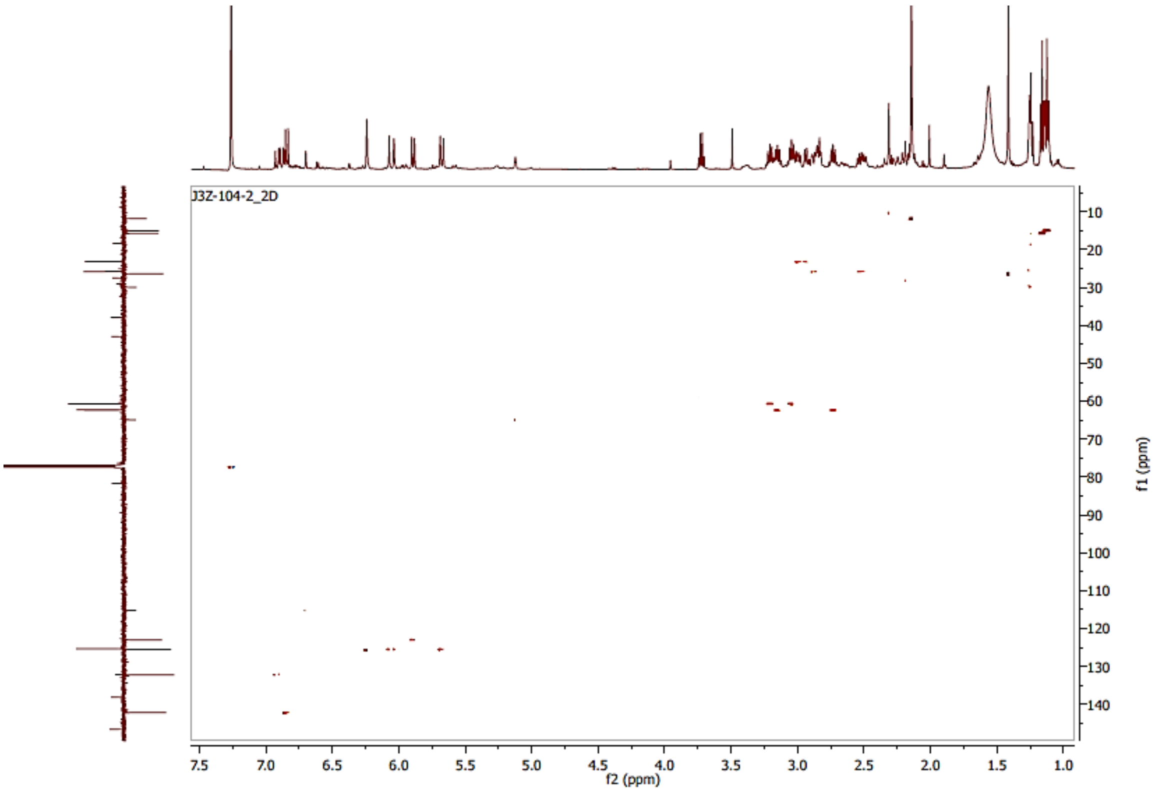

Figure S15. HSQC spectrum of compound **2a** (in $CDCl_3$).

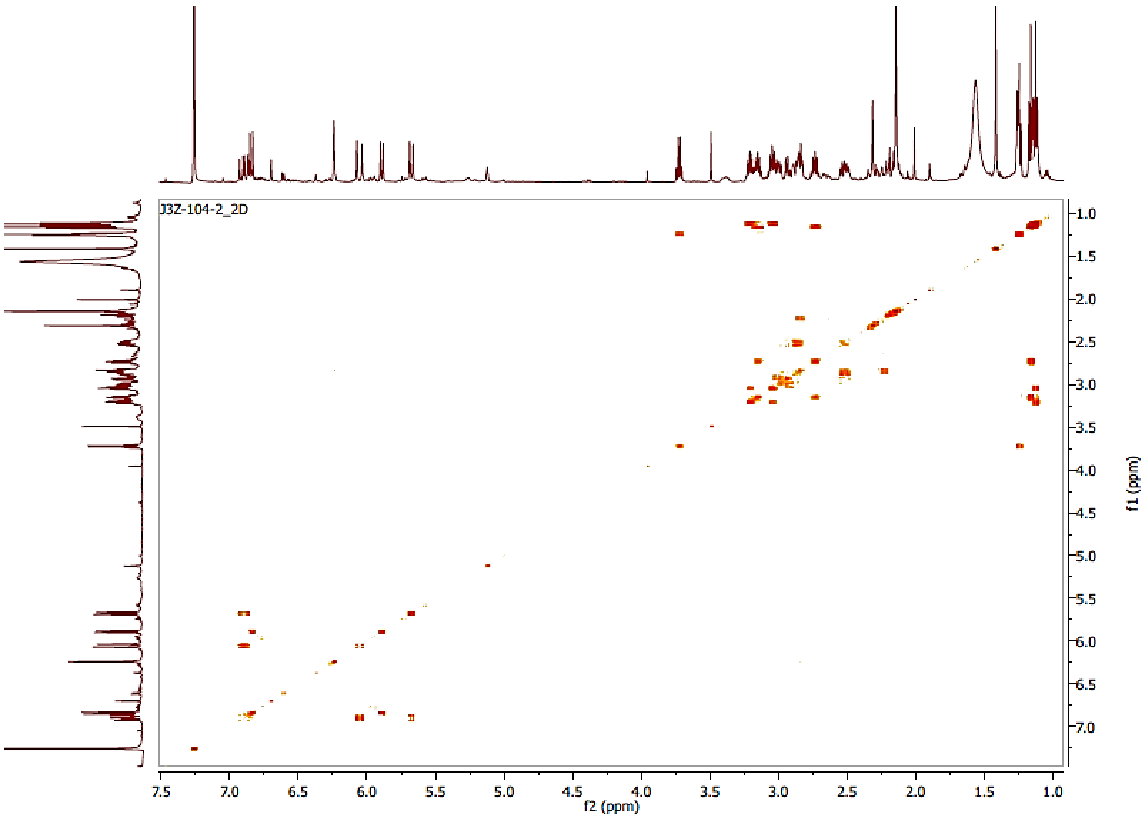

Figure S16. ^1H-^1H COSY spectrum of compound **2a** (in $CDCl_3$).

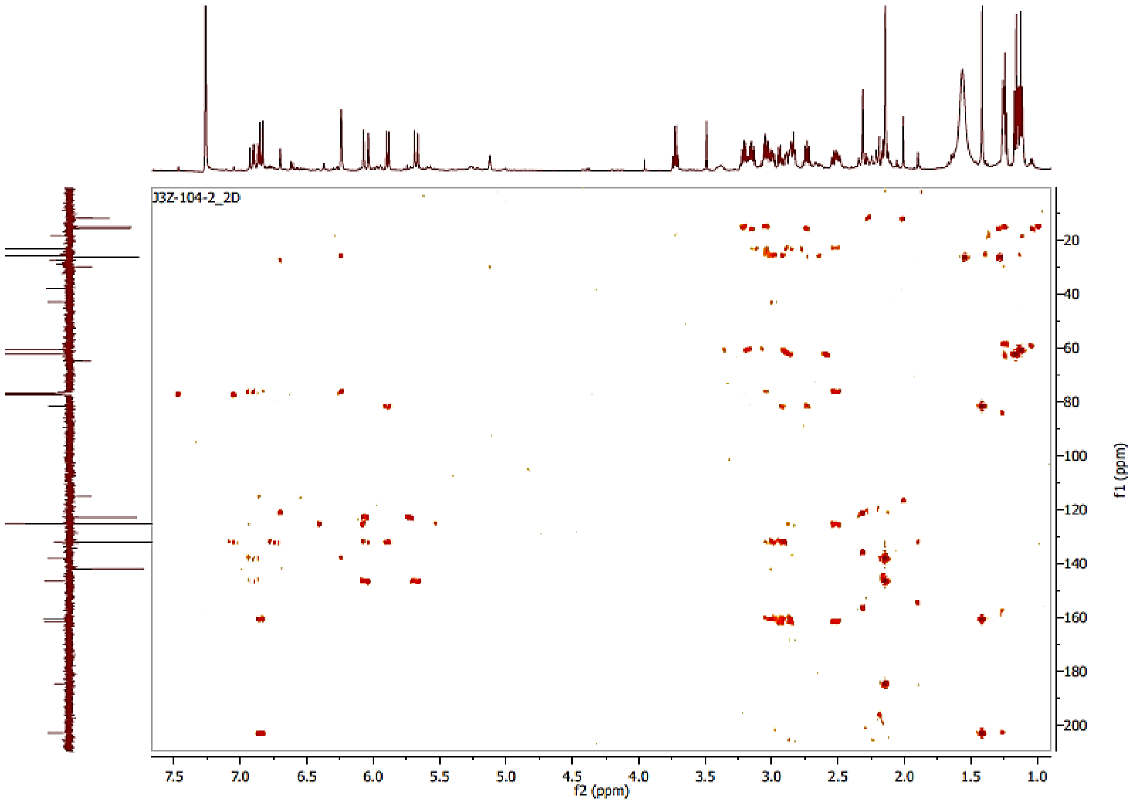

Figure S17. HMBC spectrum of compound **2a** (in $CDCl_3$).

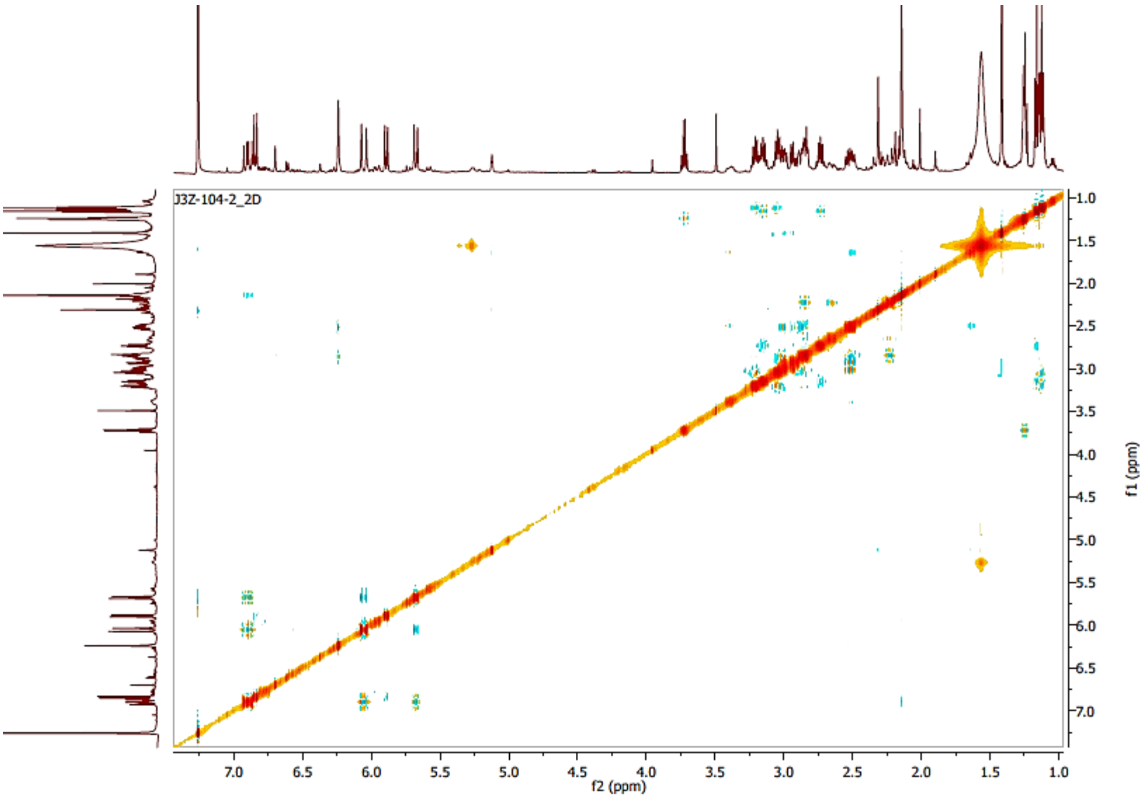

Figure S18. NOESY spectrum of compound **2a** (in $CDCl_3$).

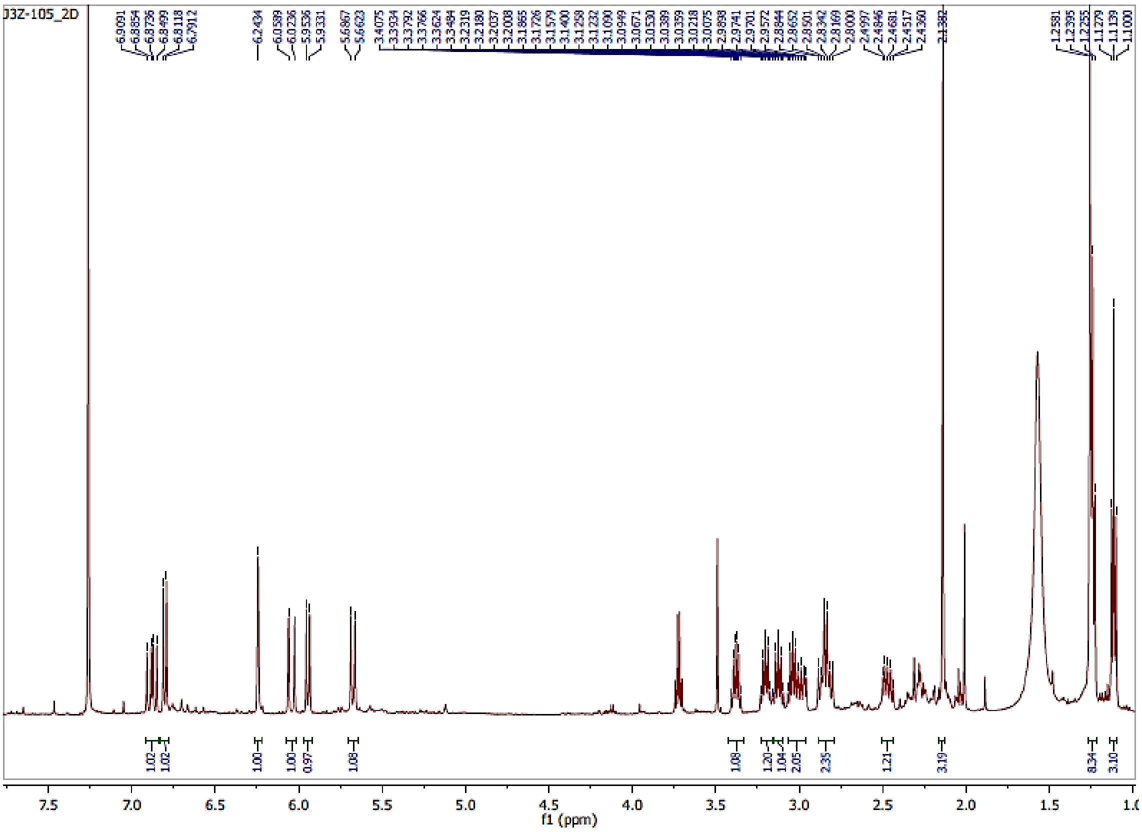

Figure S19. ^1H NMR spectrum of compound **2b** (500 MHz, in $CDCl_3$).

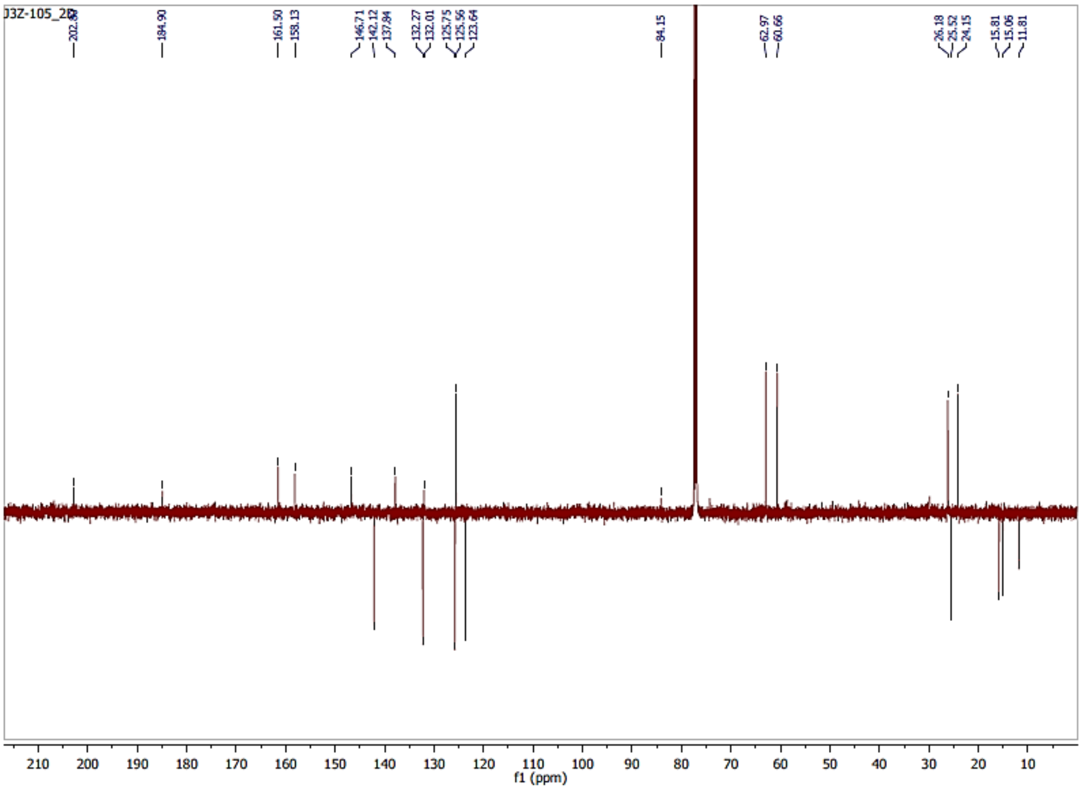

Figure S20. ^{13}C JMOD NMR spectrum of compound **2b** (125 MHz, in $CDCl_3$).

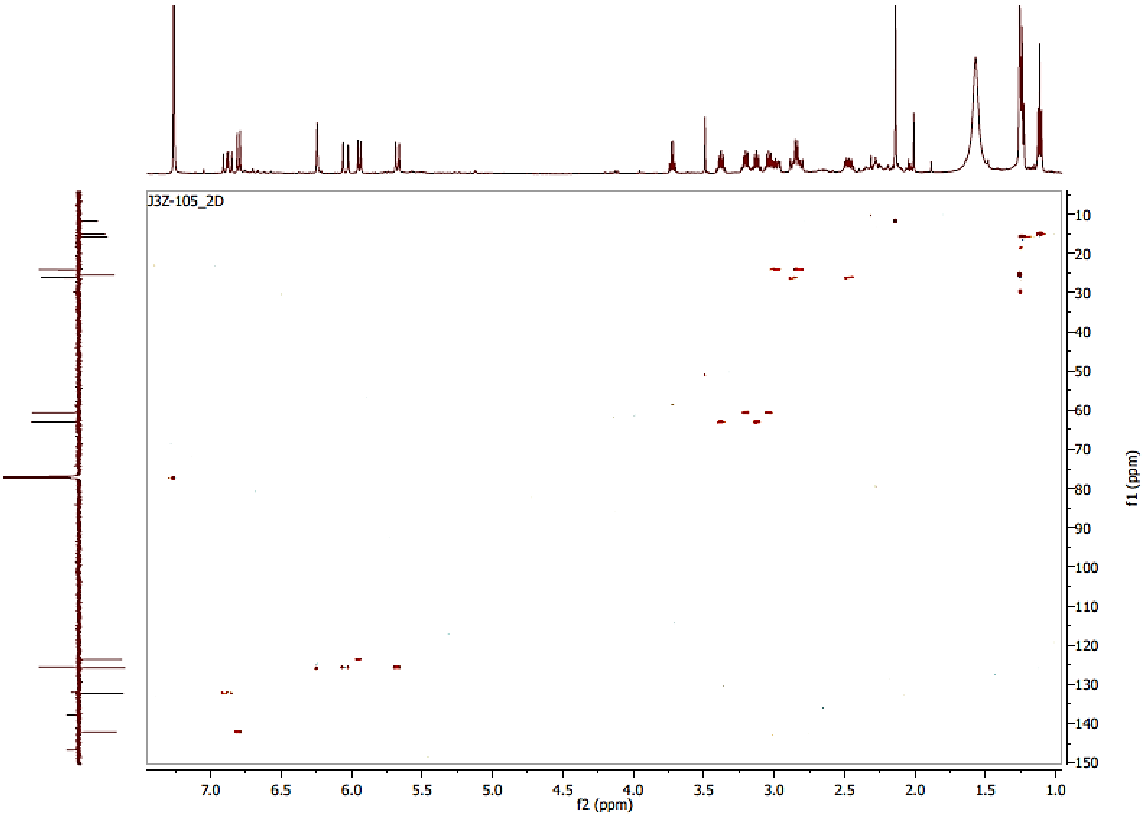

Figure S21. HSQC spectrum of compound **2b** (in $CDCl_3$).

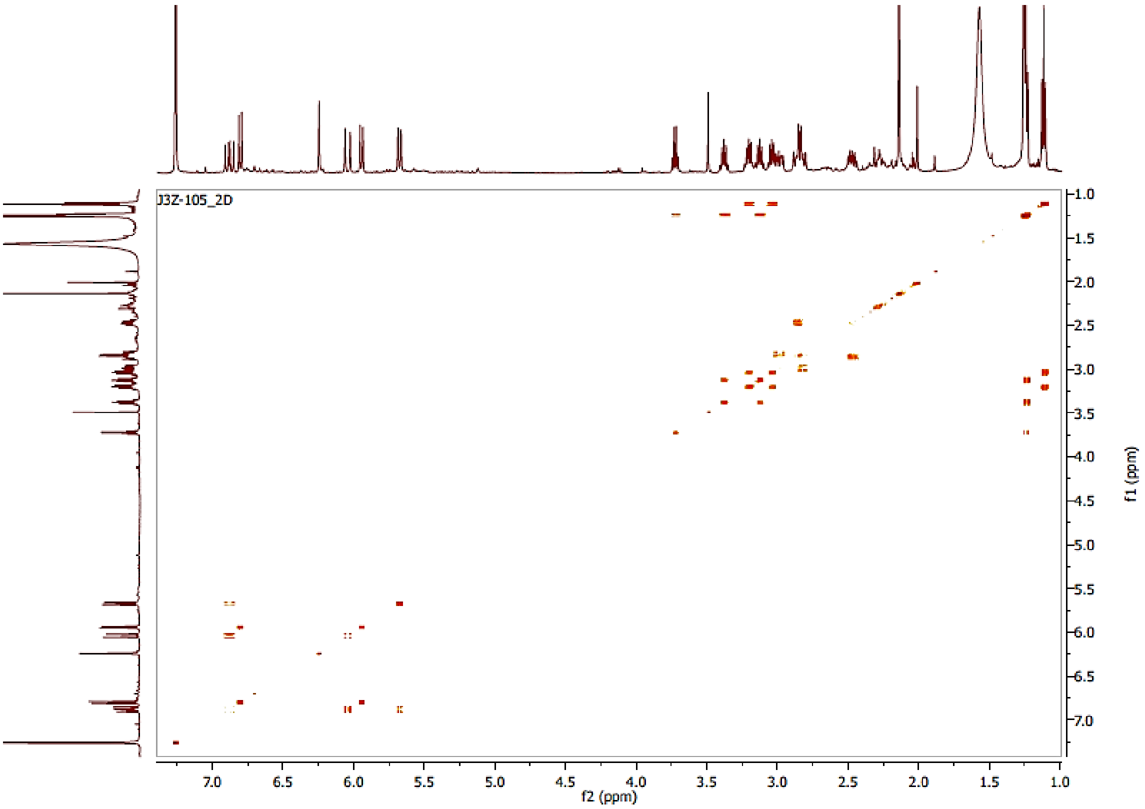

Figure S22. ^1H-^1H COSY spectrum of compound **2b** (in $CDCl_3$).

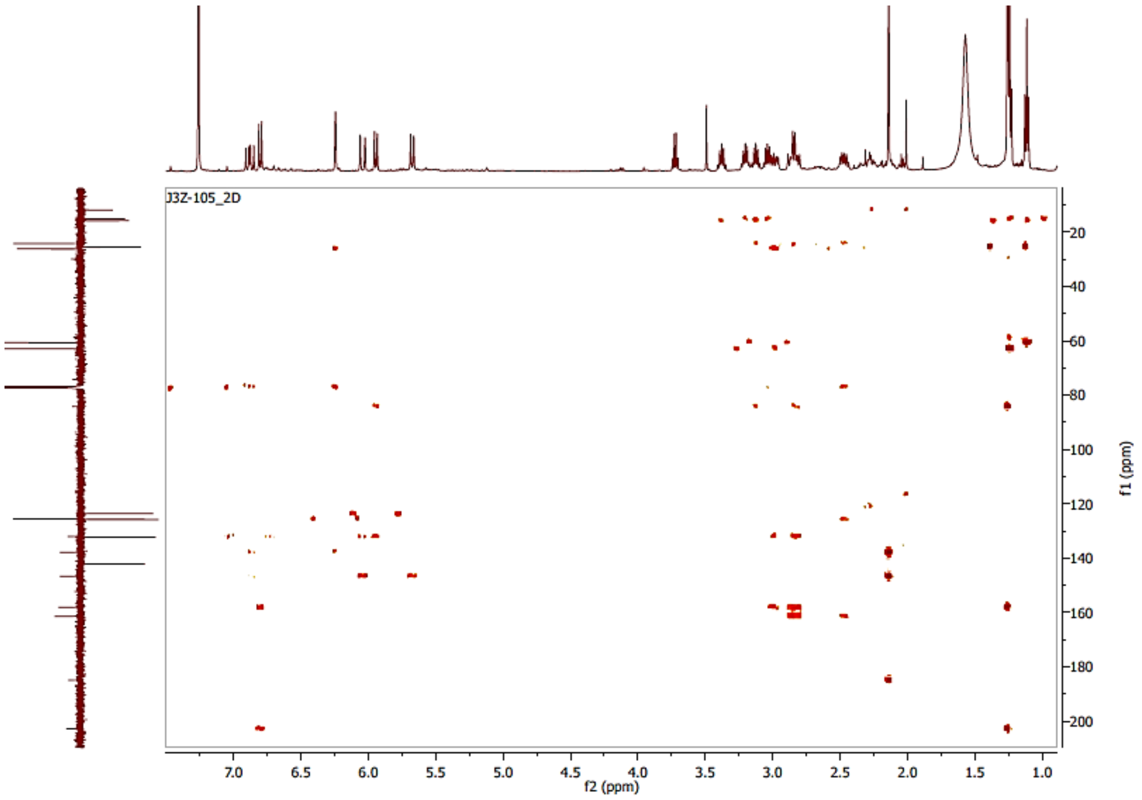

Figure S23. HMBC spectrum of compound **2b** (in $CDCl_3$).

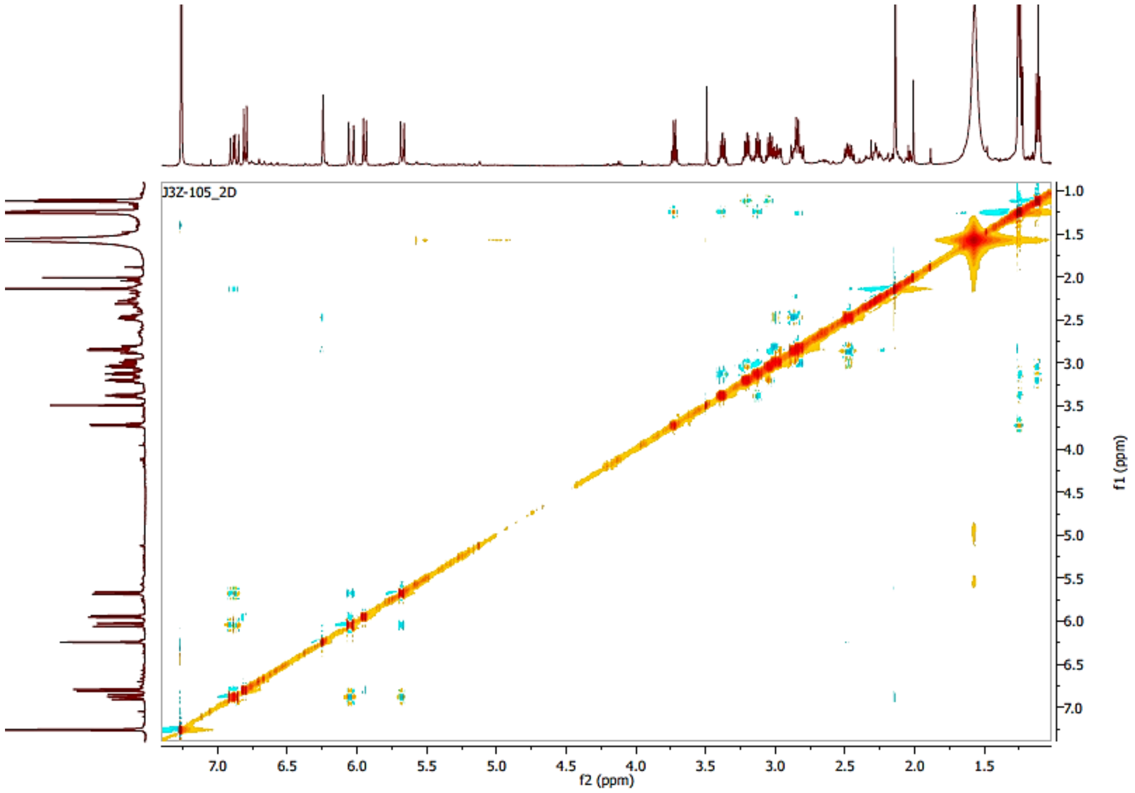

Figure S24. NOESY spectrum of compound **2b** (in $CDCl_3$).

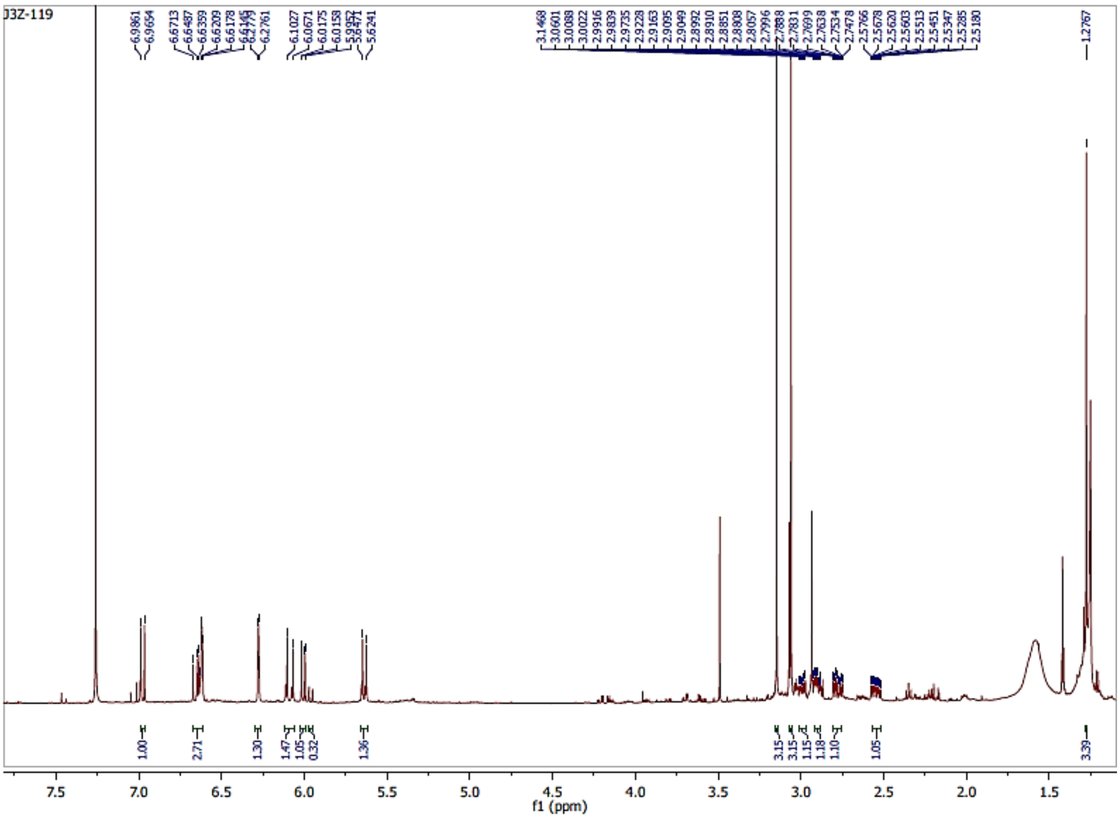

Figure S25. ^{1}H NMR spectrum of compound **3** (500 MHz, in $CDCl_3$).

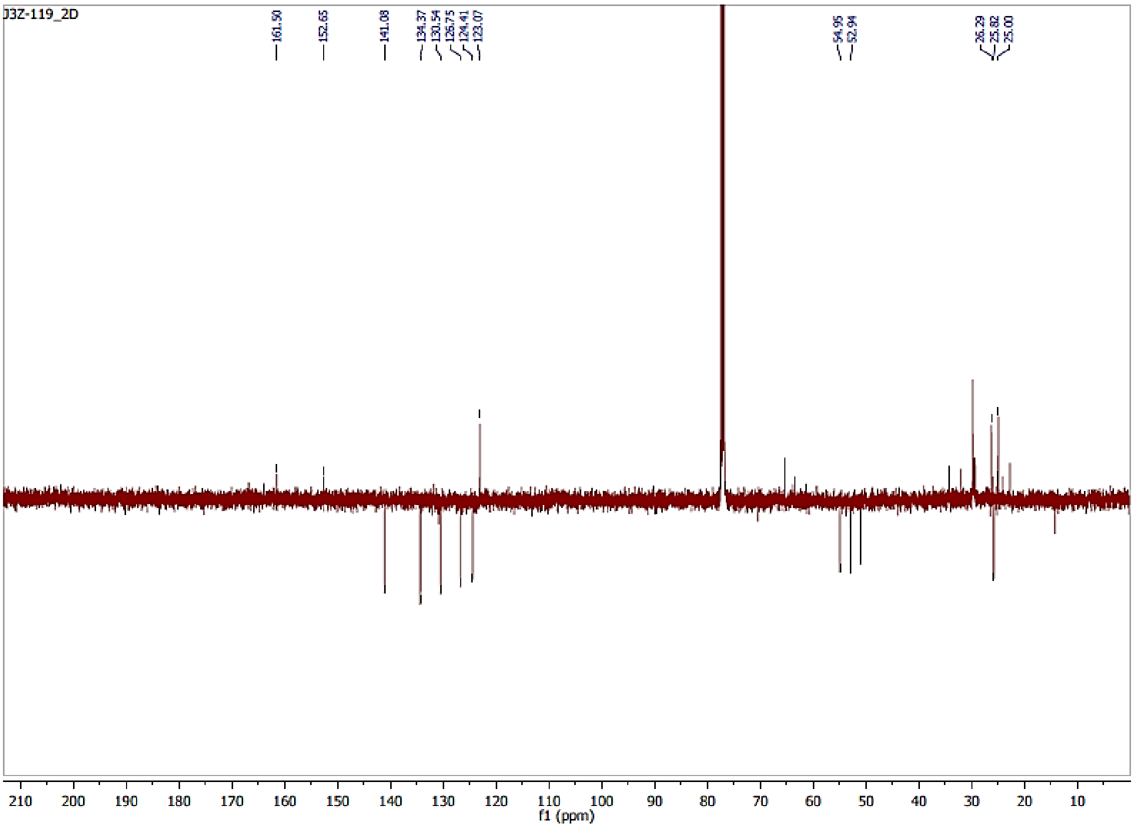

Figure S26. ^{13}C JMOD NMR spectrum of compound **3** (125 MHz, in $CDCl_3$).

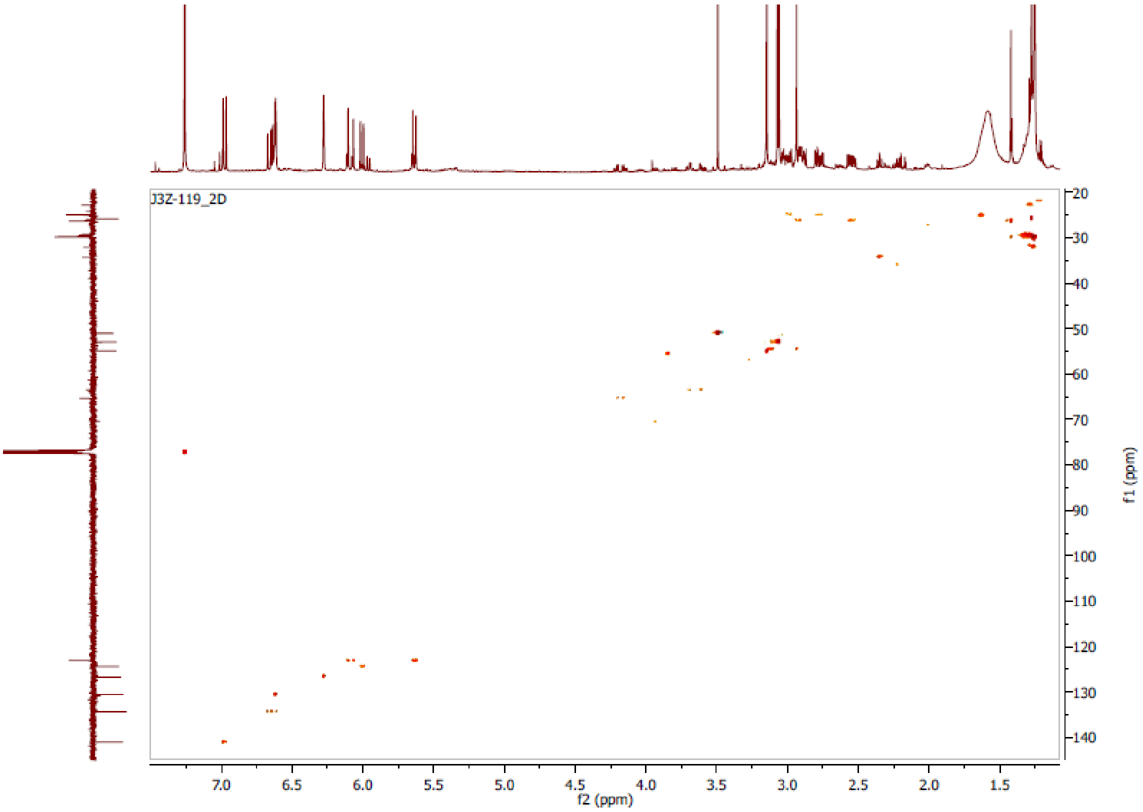

Figure S27. HSQC spectrum of compound **3** (in $CDCl_3$).

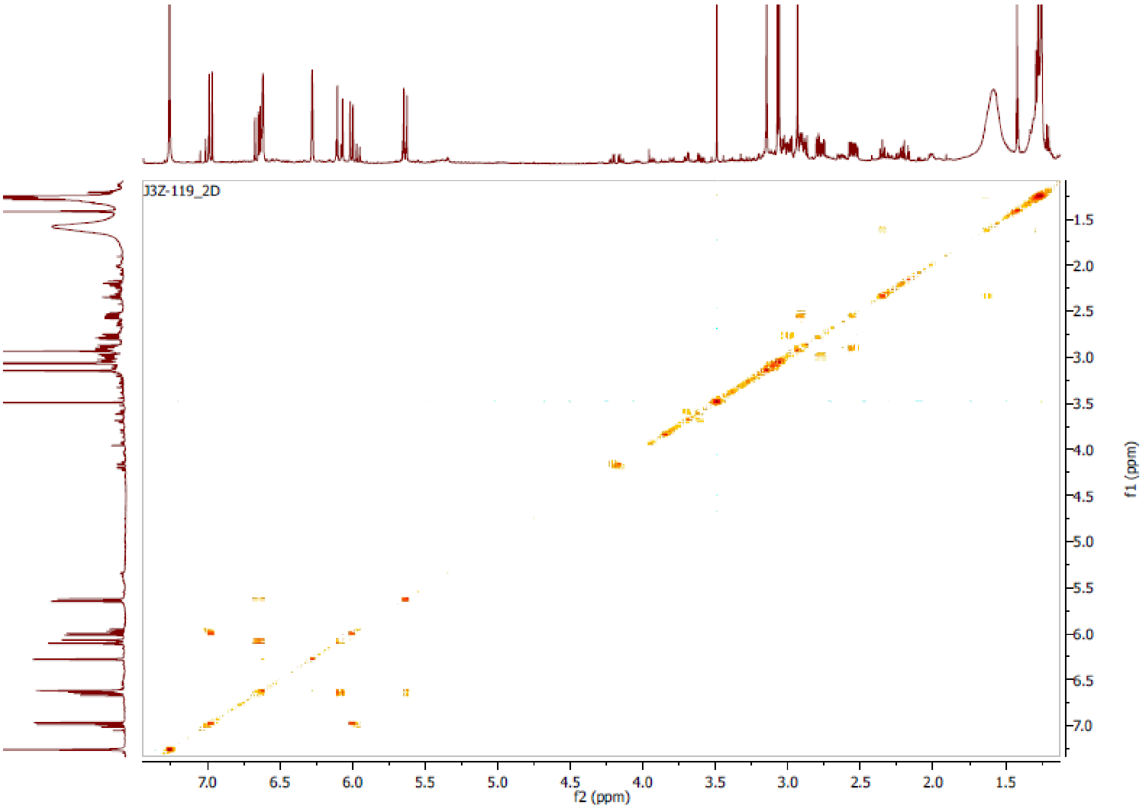

Figure S28. ^1H-^1H COSY spectrum of compound **3** (in $CDCl_3$).

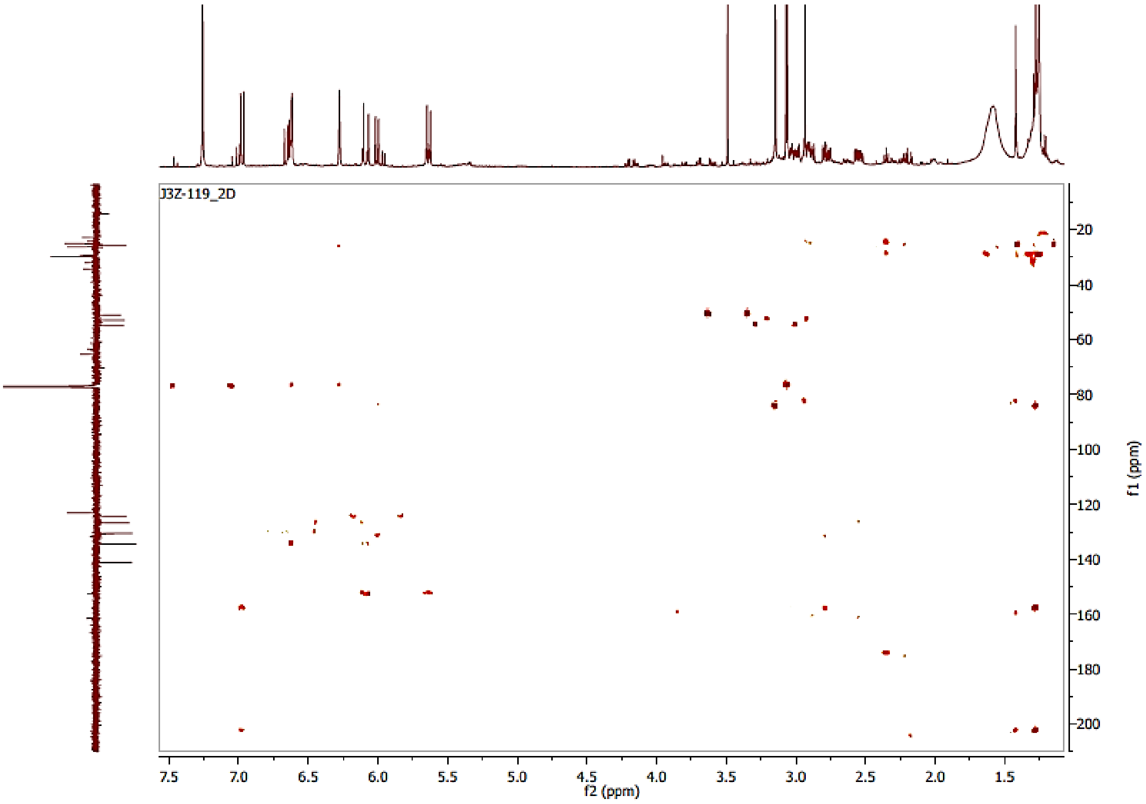

Figure S29. HMBC spectrum of compound **3** (in $CDCl_3$).

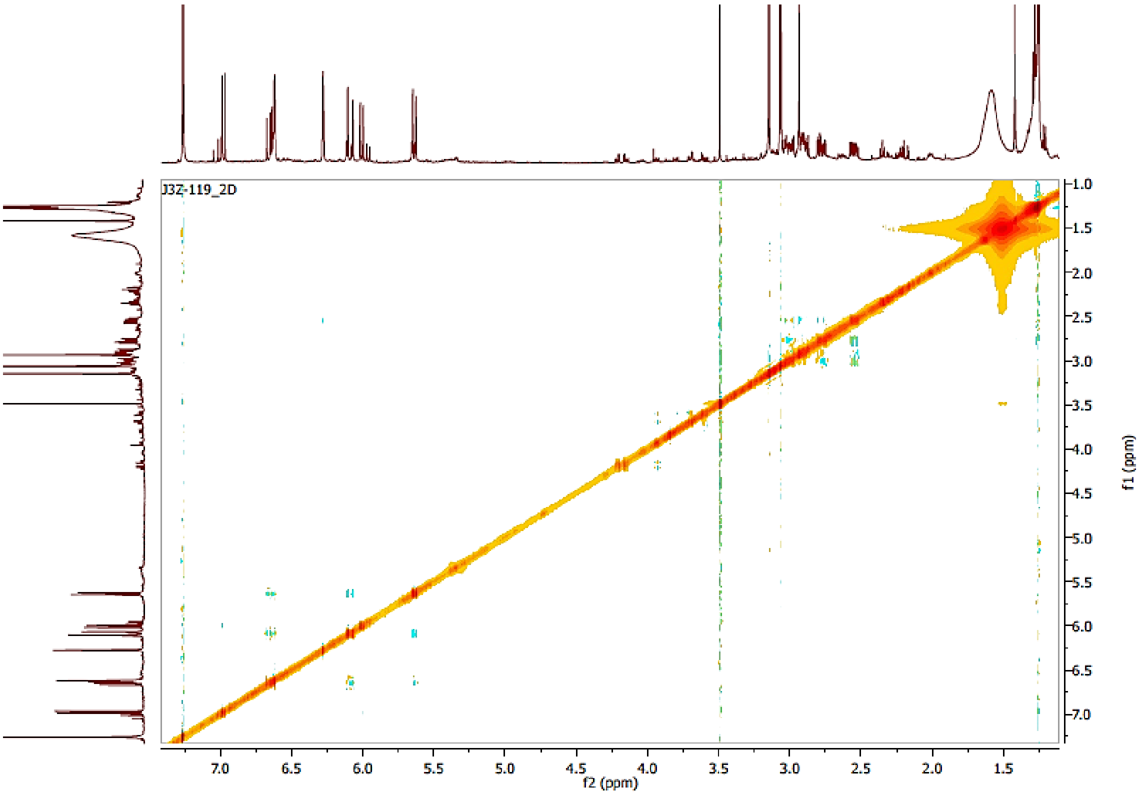

Figure S30. NOESY spectrum of compound **3** (in $CDCl_3$).

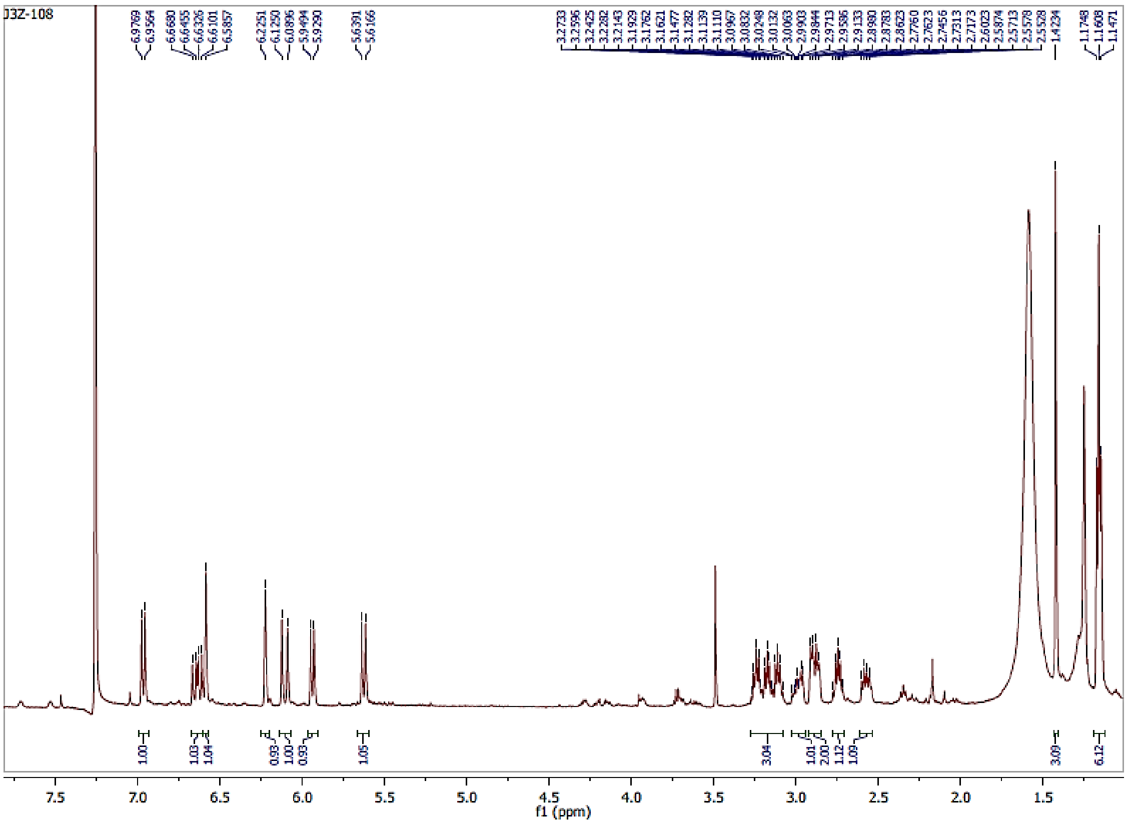

Figure S31. ^{1}H NMR spectrum of compound **4a** (500 MHz, in $CDCl_3$).

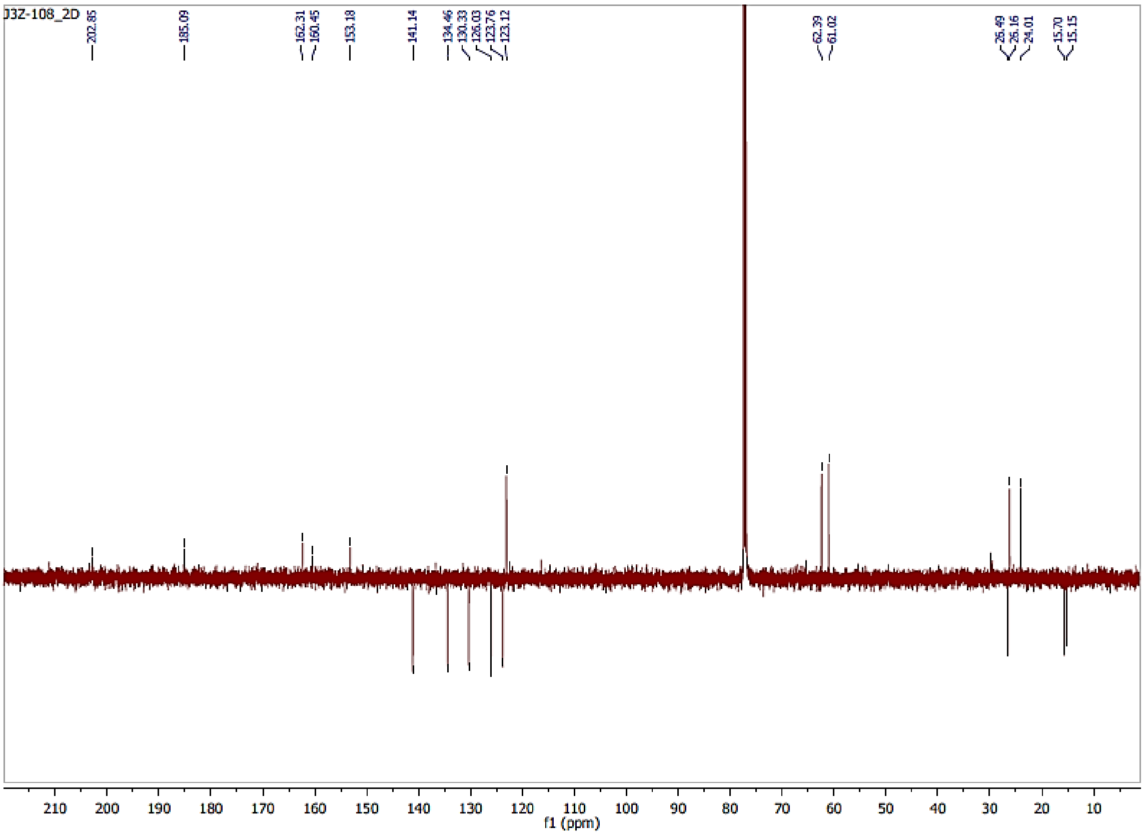

Figure S32. ^{13}C JMOD NMR spectrum of compound **4a** (125 MHz, in $CDCl_3$).

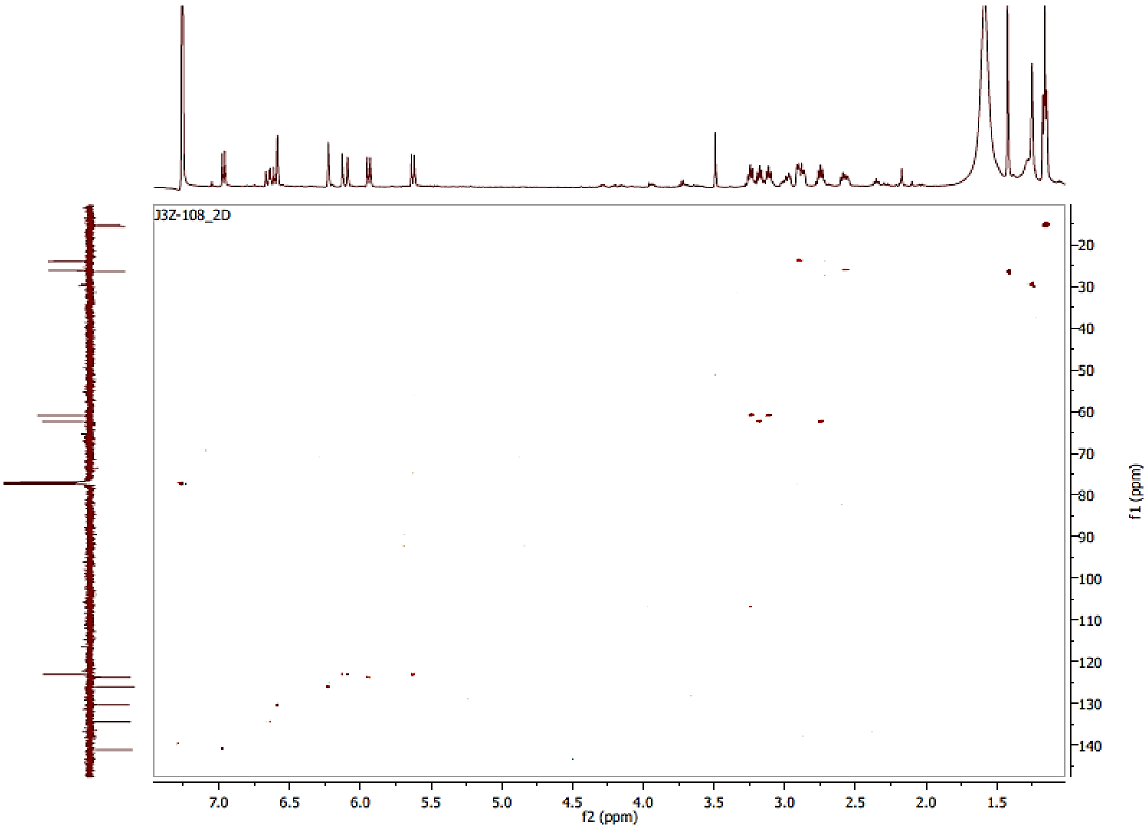

Figure S33. HSQC spectrum of compound **4a** (in $CDCl_3$).

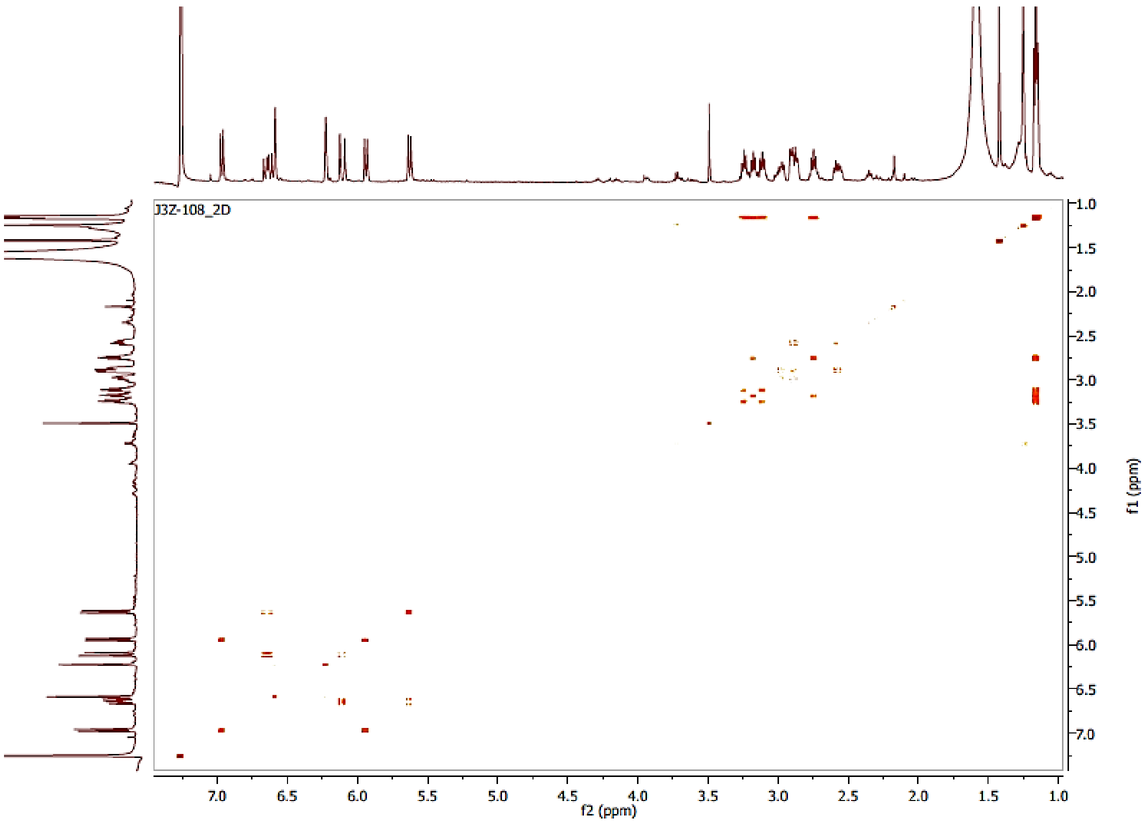

Figure S34. ^1H-^1H COSY spectrum of compound **4a** (in $CDCl_3$).

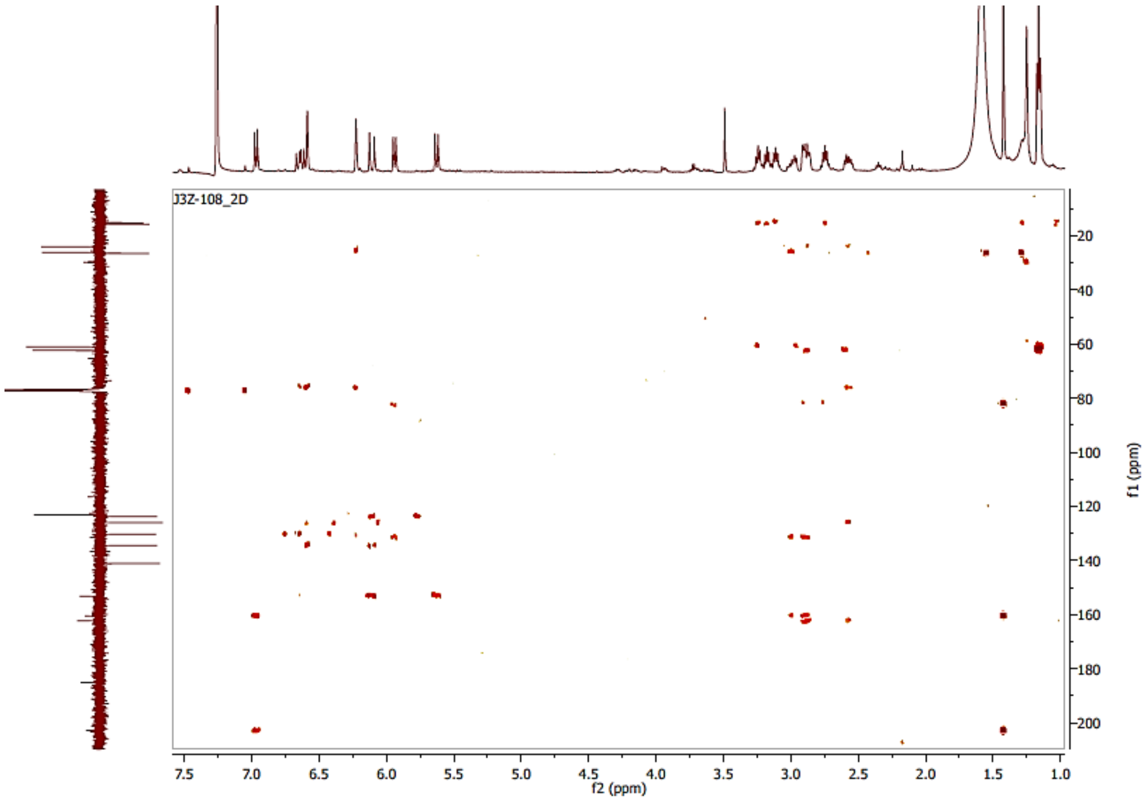

Figure S35. HMBC spectrum of compound **4a** (in $CDCl_3$).

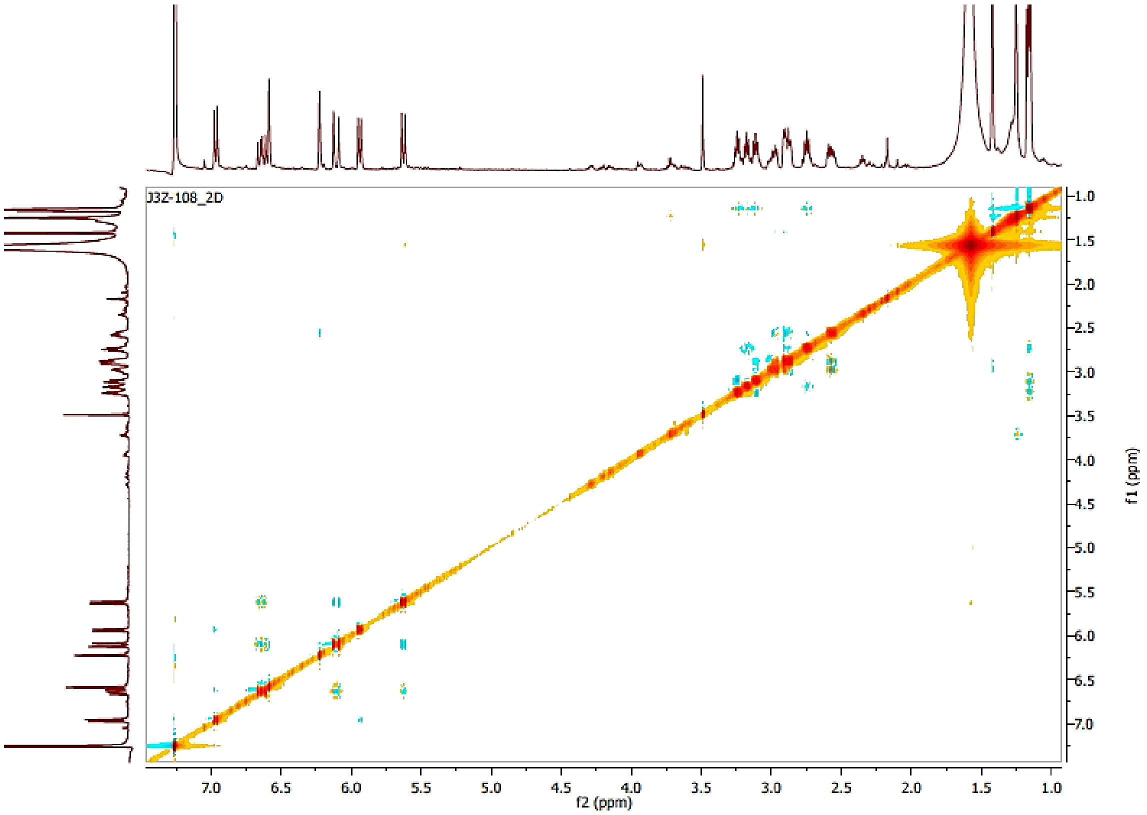

Figure S36. NOESY spectrum of compound **4a** (in $CDCl_3$).

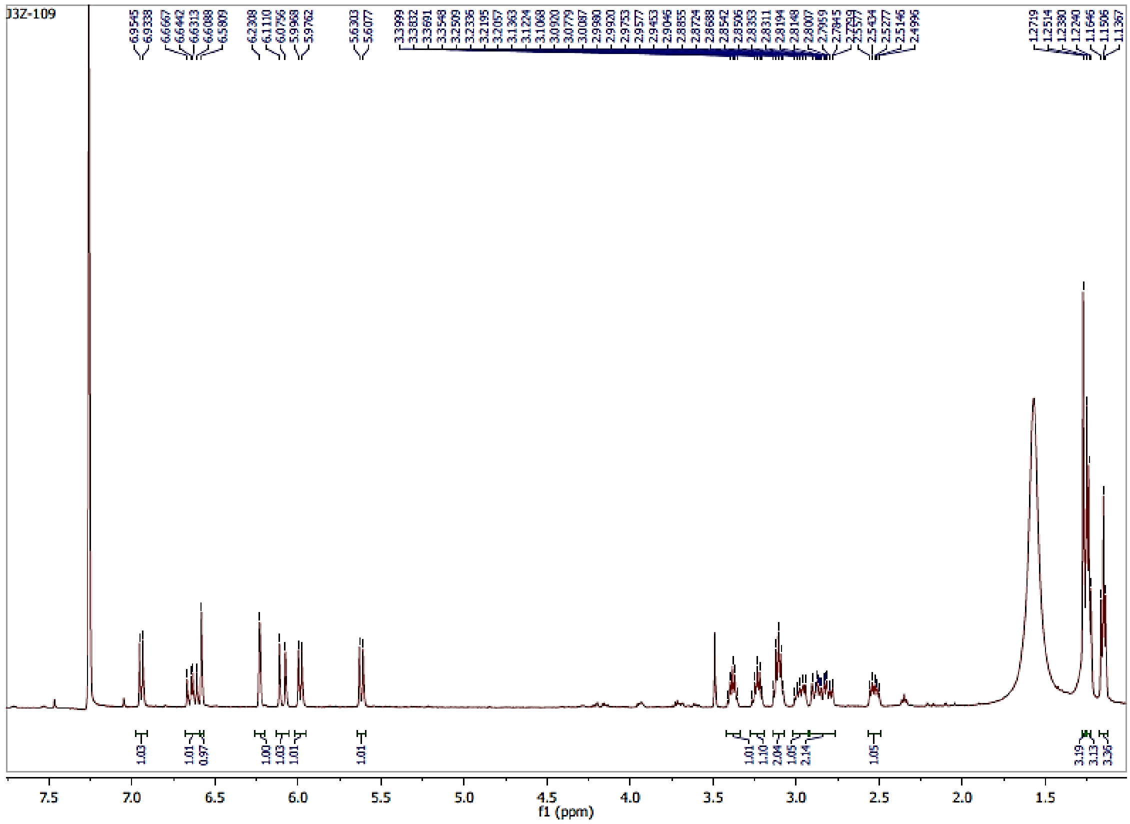

Figure S37. ^{1}H NMR spectrum of compound **4b** (500 MHz, in $CDCl_3$).

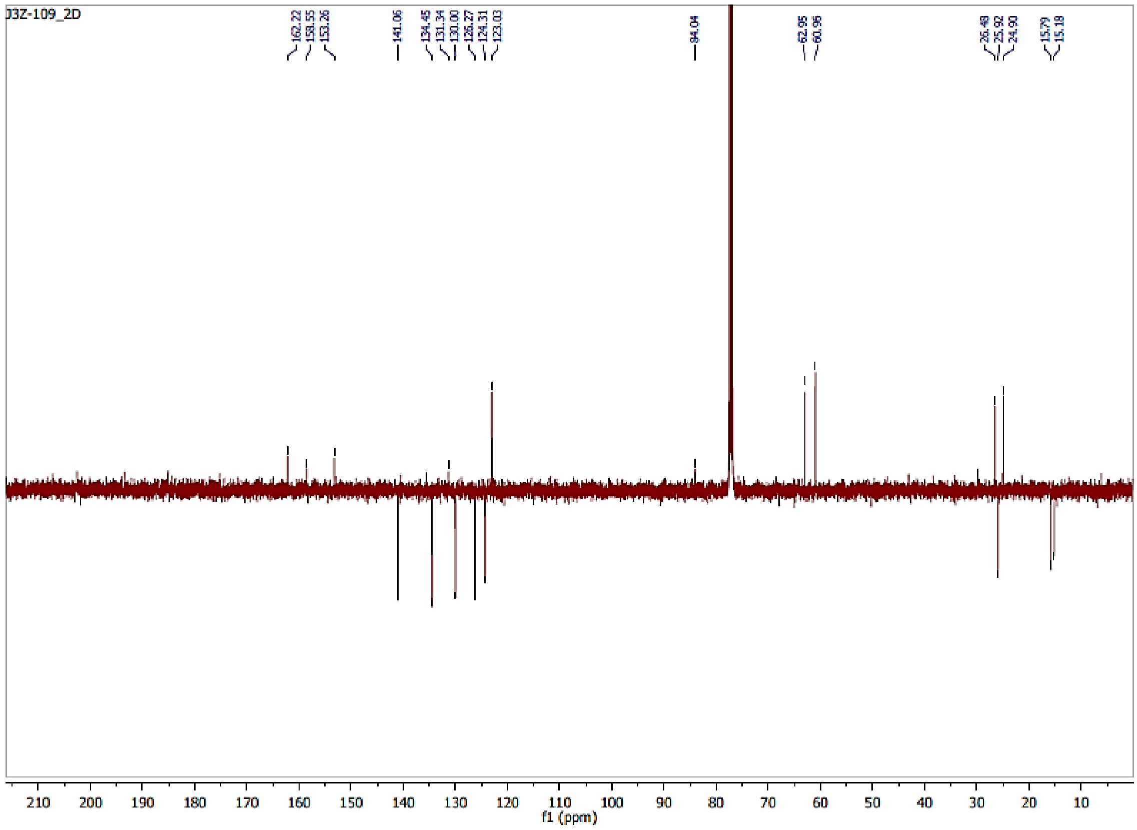

Figure S38. ^{13}C JMOD NMR spectrum of compound **4b** (in $CDCl_3$).

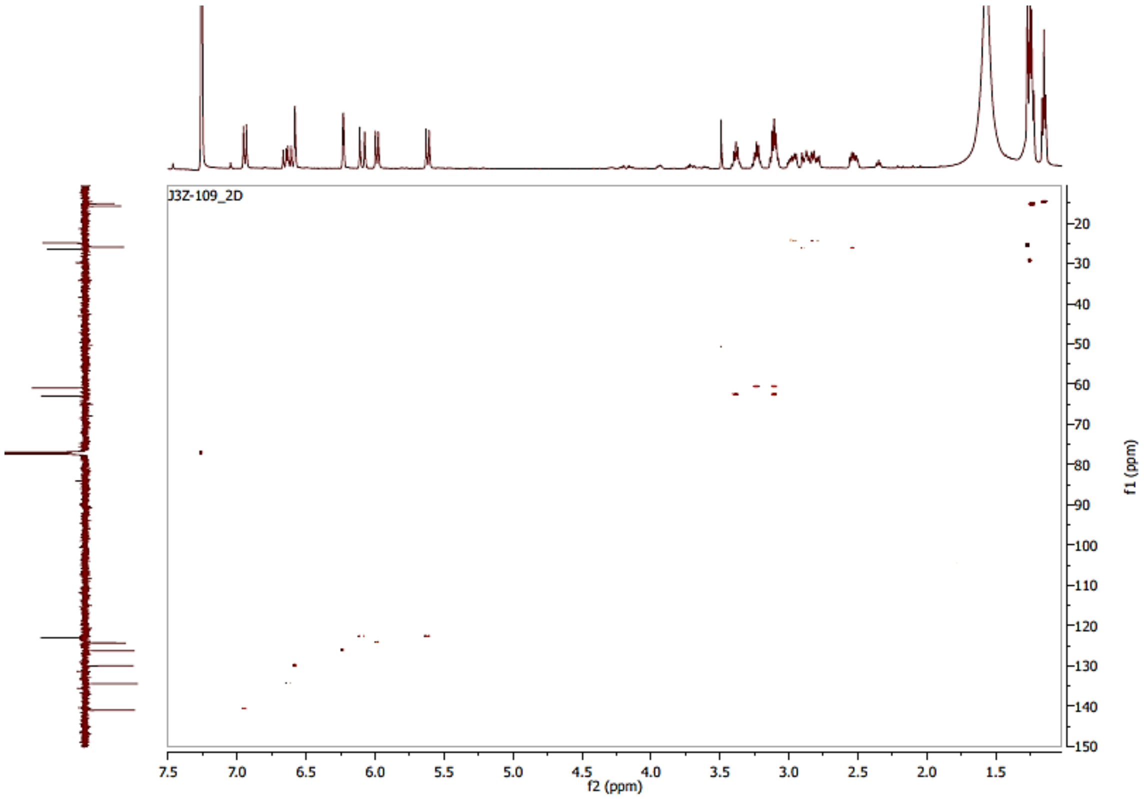

Figure S39. HSQC spectrum of compound **4b** (in $CDCl_3$).

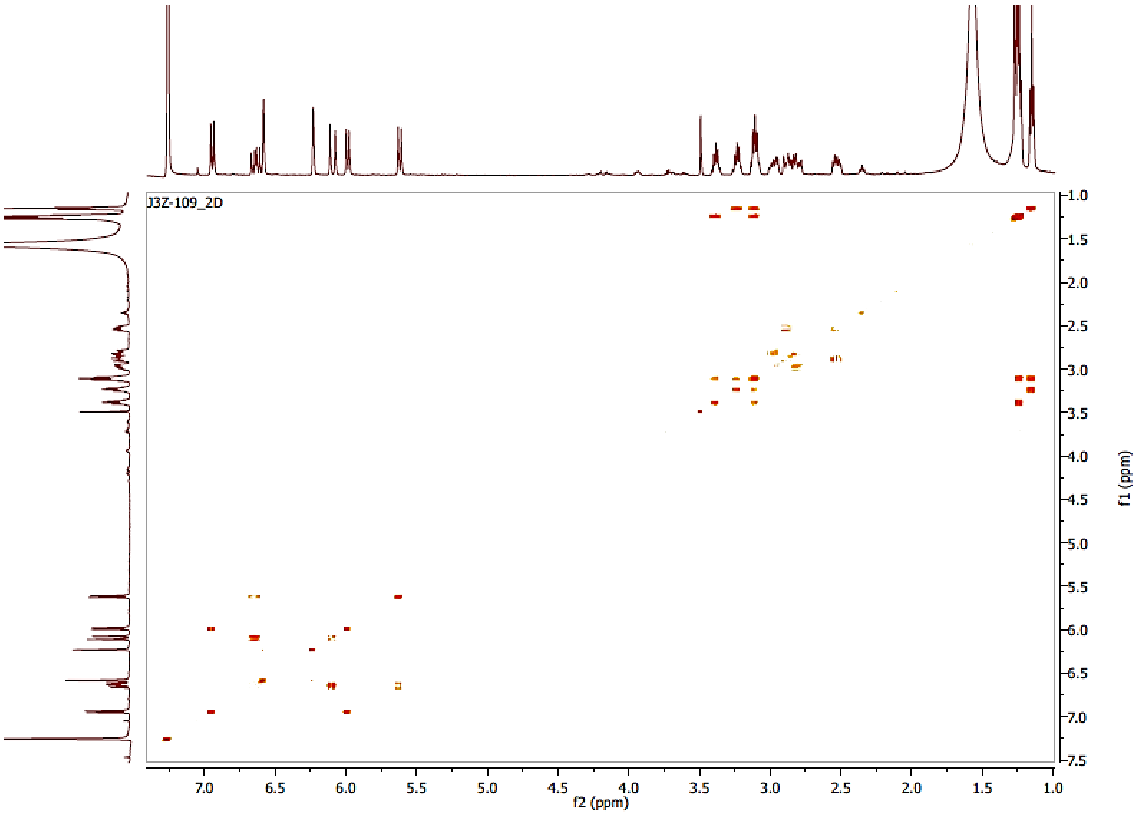

Figure S40. ^{1}H-^{1}H COSY spectrum of compound **4b** (in $CDCl_3$).

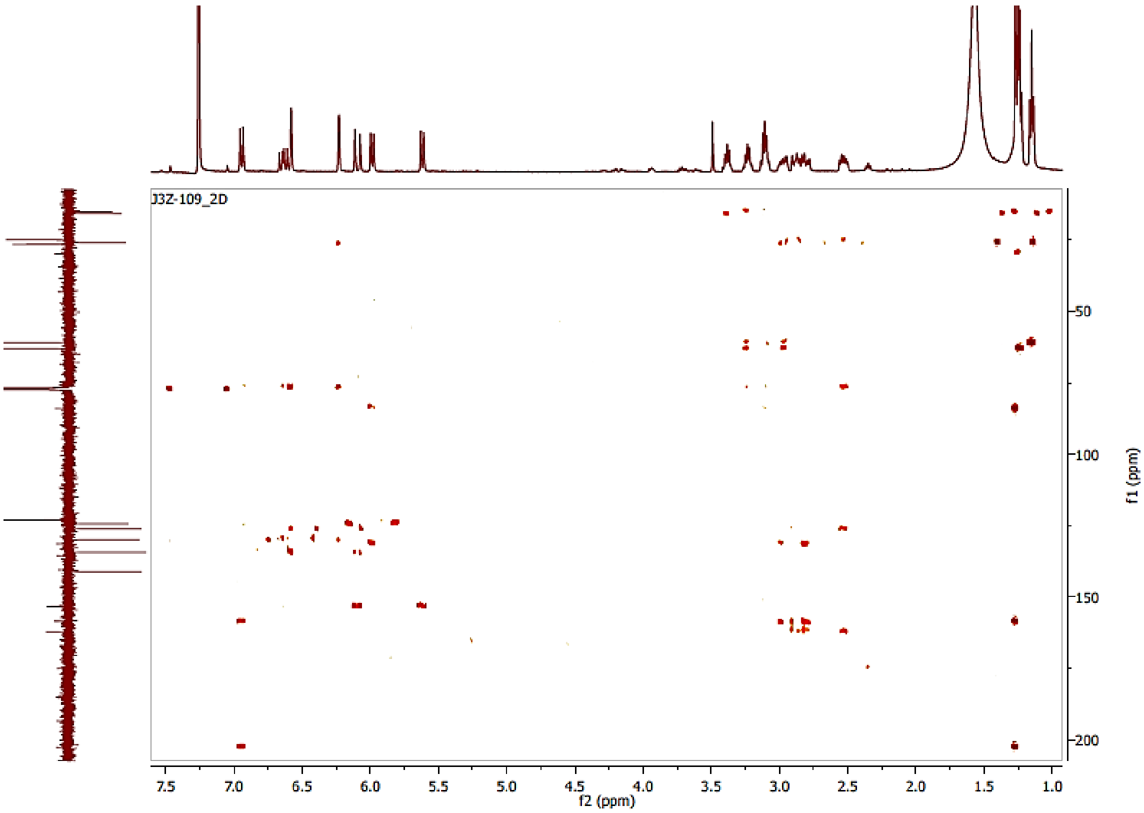

Figure S41. HMBC spectrum of compound **4b** (in $CDCl_3$).

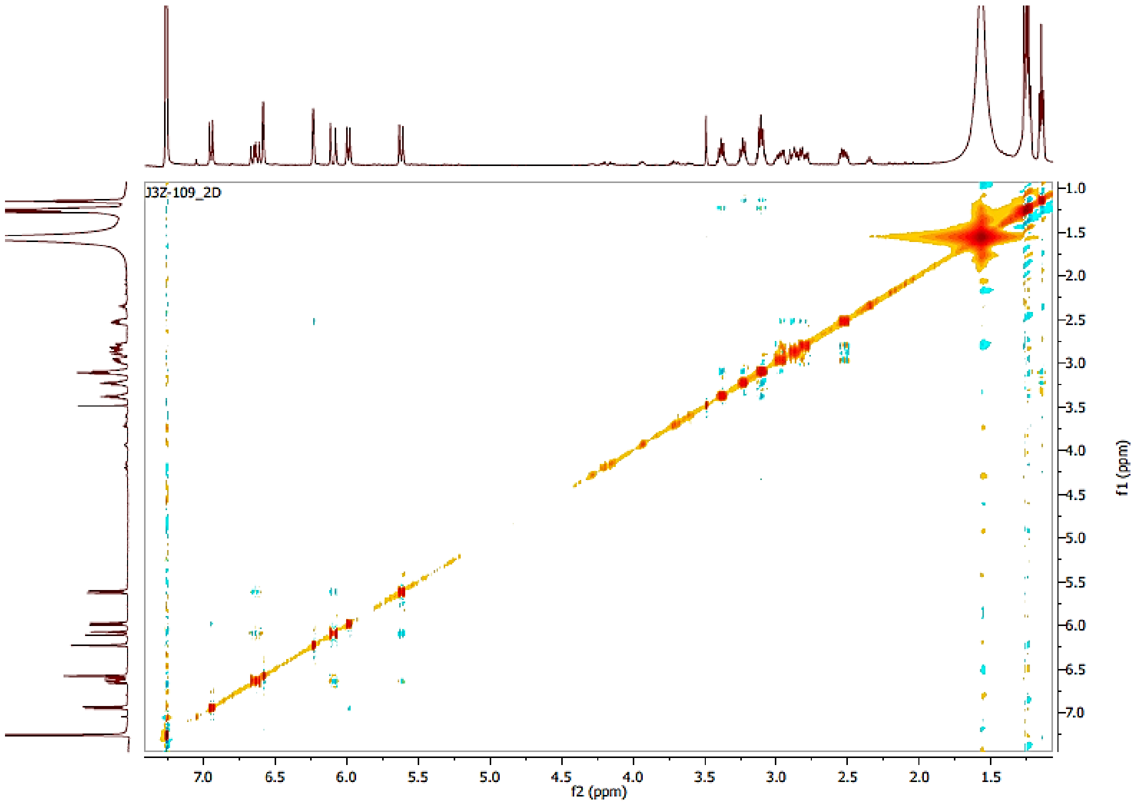

Figure S42. NOESY spectrum of compound **4b** (in $CDCl_3$).
